# Perceptions of Arab men regarding female breast cancer screening examinations—Findings from a Middle East study

**DOI:** 10.1371/journal.pone.0180696

**Published:** 2017-07-21

**Authors:** Tam Truong Donnelly, Al-Hareth Al-Khater, Salha Bujassoum Al-Bader, Mohamed Ghaith Al-Kuwari, Mariam Ali Abdul Malik, Nabila Al-Meer, Rajvir Singh

**Affiliations:** 1 Faculty of Nursing and Medicine, University of Calgary, Calgary, Alberta, Canada; 2 Department of Hematology and Oncology, Hamad Medical Corporation, Doha, Qatar; 3 Healthy Lifestyle Program, Aspetar, Doha, Qatar; 4 Primary Health Care, Qatar Ministry of Public Health, Doha, Qatar; 5 Nursing, Qatar Ministry of Public Health, Doha, Qatar; 6 Research Department, Hamad Medical Corporation, Doha, Qatar; Rudjer Boskovic Institute, CROATIA

## Abstract

**Objectives:**

In the Middle East, Qatar in particular, the incidence of breast cancer has substantially increased in recent years, and is expected to double by 2030. This diagnosis also occurs at a later stage in the disease. Early detection along with proper treatment reduces radical mastectomy and mortality rates, yet only one-third of Arab women in Qatar participate in breast cancer screening (BCS) activities of any sort. Many women in the conservative Qatari society rely on male family members for support and protection. This study investigates the attitudes and perceptions of Arab men in regards to breast cancer screening and what they see as both incentives and barriers to women’s participation in BCS activities.

**Design:**

A qualitative methodology using purposive sampling technique was chosen in order to explore participant’s attitudes, beliefs and health-related actions. Individual in-depth interviews with open-ended questions were conducted with 50 Arab men during October 2011 to May 2012. Data collection, analysis, and interpretation occurred simultaneously. NVivo 9, a qualitative data analysis software program was used to organize themes and subthemes.

**Results:**

It was found that most men understood the importance of regular BCS in early detection of breast cancer. They felt they had an important role in encouraging the women in their lives to participate in BCS activities, but were adamant that any examination must be done by a female health care professional. Few knew details about screening guidelines in Qatar, but most had a basic knowledge of some screening activities. Most indicated an interest in learning more about BC and screening activities in order to better help and inform their female family members.

**Conclusion:**

Because Arab men perceive that their opinions and support are a major factor influencing female family members’ participation in breast cancer screening, it is important that any program instituted to increase such screening participation be aimed at both men and women. More information is needed by both sexes as to the need for and benefits of regular screening activities, the techniques used, and the newly revised guidelines in Qatar. Such a program needs to be introduced in the near future in order to avert, at least partially, the expected doubling in breast cancer cases by 2030 in the Middle East.

## Background

While incidence of breast cancer in the Middle East region is lower than in other western countries, it has substantially increased in the last quarter century [1]. Furthermore, the diagnosis of breast cancer in this region often occurs at a later stage in the progress of the disease and in a higher proportion of women in their thirties and forties [1, 2, 3, 4] than in industrialized nations. Breast cancer that presents at a younger age is generally more aggressive with a possibly poorer prognosis [5, 6, 7, 8]. Later stage presentation of breast cancer is related to more prevalent use of mastectomy, with more than 80% of Arab women receiving modified radical mastectomy (MRM) [3]. It has been seen that widespread screening in countries with available treatment opportunities can reduce the need for radical mastectomies [3].

The mortality rate for breast cancer in Qatar (12.9/100,000 in 2008) [9] is higher than in other Arab peninsular countries, and BC is the most commonly diagnosed cancer for Qatari women. In 2012, in Qatar the estimated age standardized breast cancer incidence rate was 46.1 per 100,000 women, whereas other cancers fell below 7.1 per 100,000 except for colorectal cancer (15.5 per 100,000 women) [10]. As people in Qatar continue to adopt lifestyles that put them more at risk for cancer, such as smoking, consumption of foods rich in saturated fat and with a high caloric content along with reduced physical activity, the incidence of diagnosis of breast cancer in Qatar is expected to increase by 60% by 2020 [11] and to double by 2030 [12].

The State of Qatar provides free or heavily subsidized, high quality medical care and facilities for all residents and BCS is made available through female-staffed clinics with diagnostic equipment to facilitate screening. Early detection of breast cancer through BCS along with proper treatment has been shown to reduce mortality rates by 25–30% [13, 14]. The five-year survival rate for detection at Stage 0 is 93% and at Stage 4 is 15% [15], yet the rate of participation in breast cancer screening activities by Arab women in Qatar is low. Recent research in Qatar by Donnelly and colleagues shows that only one-third of 1063 Arab women participants participated in BCS activities [16]. Donnelly et al. study also explored what Arabic women in Qatar knew about BCS. It was found that, although 90.7% of women interviewed knew of breast cancer, only 7.6% participants were assessed with having basic knowledge of all breast cancer screening activities. It was also found that 94% of the women interviewed would have a mammogram if their doctor suggested it and participation in other screening activities was highly dependent on physician recommendations. Yet only 24.4% reported that their doctors ever discussed breast cancer with them or recommended BCS [16].

The study cited above also found that getting information about breast cancer and BCS from any source significantly increased participation in screening activities. Because Qatari society is fundamentally conservative, many Arab women are reliant on husbands, brothers and fathers for support and protection. For this reason, any program promoting BCS in Qatar needs to explore men’s perspective as well as women’s perspective [3]. Asking men to encourage their female family members to participate in BC screening activities has been shown to be effective strategy. Since the recent introduction of such a national campaign in Bahrain in 2005, there has been a significant rise in diagnosis of breast cancer at an early stage [17].

The first part (quantitative) of Donnelly et al’s study [16] explores BCS activities amongst Arab women in Qatar looked at women’s BCS awareness, knowledge and participation. The second part of their research project (qualitative) reported here looks at what Arab men perceive as both facilitators and barriers to BCS and what information would help them to better assist their female family members to engage in BCS.

### Theoretical frameworks

#### Ecological perspective

This exploratory qualitative research is based on the theoretical foundation of an ecological conceptual framework, which refers to the interaction between individuals and their physical and socio-cultural environment [18]. Based on this conceptual model, individuals’—Arab womens’ health care behaviour is influenced by their physical environmental variables, intrapersonal variables, and other social determinants of health [19, 20, 21, 22]. To facilitate behavioural changes in individuals, efforts should be directed towards altering the unfavourable and fostering the favourable factors that influence individual’s life and health care practice [21, 23].

Qatar has witnessed rapid change in many aspects of life. Rapid growth and changing environmental and social conditions have affected the prevalence and patterns of cancer. Thus, to promote breast cancer early detection, we investigated how environmental factors and other social determinants of health influence Arab women’s BCS. Family dynamic and gendered social relationships influence Arab women’s health care choices and practices, thus we also investigated those factors to identify ways in which it fosters and/or hinders women’s BCS practices from both women and men’s perspectives. Because the population of interest is Arab women and men who come from different ethno-cultural backgrounds from the Middle East society but are living in Qatar, exploring the influence of socio-cultural factors on their perspective towards health care behaviours and practices is essential to developing an effective and culturally appropriate health promotion program that will be accepted by the both women and men of Qatar. Therefore, we combine an ecological conceptual framework with Kleinman’s Explanatory Model of health and illness to guide our research.

#### Kleinman’s explanatory model of health and illness

Kleinman states that, “[people’s] beliefs about sickness…including their treatment expectations …affect the way individuals think about and react to sickness and choose among and evaluate the effectiveness of the health care practices available to them" [24]. He also theorizes that a given society’s attitude towards and use of health services is shaped by the way in which its members conceptualize health and illness and their cultural beliefs, values, behaviours, and expectations regarding treatments. Thus, Arab women and men’s explanatory models of health and illness are derived from their knowledge, beliefs and values, which are informed by their specific socio-cultural backgrounds. These knowledge and backgrounds provided individuals with an explanation for causes of sickness, symptoms, pathology, course of illness, and expectations of treatment [24]. For many Arab women, health care practices and behaviour are very much influenced by their relationship with spouses and family members within their specific social and cultural context, therefore, it is important that information be gathered from both women’s, men’s, and health care provider’s perspectives.

One of the major deterrents of client compliance, satisfaction, and appropriate use of health care services was the difference between explanatory models of recipients and providers of health care [24, 25]. Thus, providing effective health care requires that providers be able to elicit and recognize recipients’ beliefs and values with respect to their understandings of illnesses and treatments, and to negotiate these differing perspectives [24]. We have reported the health care providers’ perspective in our previous paper [26].

Conflicting health and health care perspectives, coupled with cultural insensitivity, will lead to a relationship and communications breakdown between the client and his or her health-care provider. Culturally tailored interventions that target culture-specific psychosocial barriers, delivered by community members, is an important consideration for health care providers when planning a BCS program. For these reasons, Kleinman’s view and his emphasis on understanding the explanatory of individuals’ health and illnesses, are appropriate guide of this research study.

Associated with the recognition of breast cancer as a pressing health care issue, and barriers to early detection by engaging in breast examinations, are cultural attitudes toward gender roles and expectations, and sexuality among both women and men. Breast examinations require some degree of openness about examining of the women’s body. In some cultures, discussion of breast and its examinations is considered taboo because it is associated with sexuality, and breast cancer itself creates social stigma for women [27, 28]. Bener and colleagues [29, 30] found that in some conservative Middle East areas, access to mammography clinics may be hindered if women are not allowed to drive or travel alone without a male family member. In addition, women are at risk of discovering breast cancer at the late stages if they are not comfortable raising issues of breast lumps and breast examinations, if their male relatives are not supportive of, or object to, breast cancer examinations. The above information emphasizes the importance of including men in health promotion messages about breast cancer screening, so that they can encourage and support their wives’ and female relatives’ decision to go for mammography [3]. It points to the realization that to effectively reduce breast cancer’s morbidity and mortality rates by early detection, we need to promote breast cancer screening activities in ways that are culturally appropriate and acceptable to not only Arab women, but also Arab men. Therefore, we investigated factors that influence Arab women’s breast cancer screening activities from the Arab men’s perspectives. In this paper, we report findings from in-depth interviews conducted with Arab men to (a) gain insight on their knowledge regarding breast cancer and its screening for early detection and treatment of breast cancer; (b) their view of the facilitators and barriers for breast examinations (CBE, BSE, and mammogram); and (c) what information they want to have in order to increase awareness of breast cancer and to assist Arab women to engage in breast cancer examinations. An exploratory qualitative research design using in-depth interviews as the method of data gathering provided more detailed contextual information that underlies participant’s perspectives. In a cross cultural study where a sensitive topic such as breast cancer and breast examinations are investigated and where different language and cultural perspectives are prevalent, conducting individual in-depth interviews enabled research participants to describe their experiences and the meaning they attribute to these experiences using their own words.

## Methods

Ethics approval for this research study was obtained from the Hamad Medical Corporation Research Committee (Ethics Approval Reference No: RC/1744/2010), the Qatar Supreme Council of Health (Ethics Assurance No: SCH-AUCQ-050), and the University of Calgary’s Conjoint Health Research Ethics Board (Ethics ID: E-23551). Because little research has been conducted in Qatar, participants might not be familiar with the research process. Careful explanation of the project was exercised by the research team. To make the information accessible to everyone, all the information from the project was translated into Arabic and was available in both Arabic and English. Prior to the start of the study, communities were made aware of the study through announcements in local newsletter, newspaper, community- based organizations, and by word-of-mouth. Project staff connected with all sites at the start of the project. An introductory letter written in both Arabic and English was sent to the hospitals and community clinic sites. This letter explained the purpose of the study, its objectives, research question, and participants’ recruitment criteria. Formal and informal presentations were made to all staff at the hospital and community health clinics at the start of the project and posters in Arabic with the project information were distributed prior to the start of the study. Written and oral consent to participate in this study was obtained from each participant. Throughout the interview, permission to continue with the process was also obtained intermittently and orally. Prior to conducting an interview, each participant was given an explanation of the study and informed of his rights according to the standard interview guideline. Participants were assured that all information would remain confidential and interview questionnaires were stripped of identifying information to preserve confidentiality. At the end of the interview each participant was provided a small reimbursement of $50 for his time and effort. According to the Council for International Organizations of Medical Science, compensating for participants’ inconvenience and time spent is acceptable practice [31]. All participants’ names used in this paper are pseudonyms. Pseudonyms are used in referring to comments by the participants.

### Data collection

Qualitative methodology using individual in-depth interviews and semi-structured questionnaire with open-ended questions were conducted with 50 Arab men. In-depth interviewing is "a data gathering technique used in qualitative research when the goal is to collect detailed, richly textured, person-centered information from one or more individuals" [32]. We chose this method of data collection for the study because it is particularly well suited to exploring participants’ attitudes, beliefs and health related-actions. In a cross-cultural study where a sensitive topic such as breast cancer and breast examinations are investigated and where different language and cultural perspectives are prevalent, conducting in-depth interviews enables research participants to describe their experiences and the meaning they attribute to these experiences using their own words [33]. Purposive sampling technique [34] was used to recruit men participants from seven different research sites in urban and semi-urban Qatari hospital settings and community health clinics. Inclusion criteria were: men who were 30 years of age and older, spoke Arabic and they should have lived in Qatar for 10 or more years. Experienced and especially trained bi-lingual (Arabic and English) male nurses and nurse students conducted interviews in Arabic. The interviews were audio-taped, translated into English, and transcribed verbatim.

Interview questions were asked to gain Arab male perspectives regarding women receiving breast examinations and what they perceive to be the needs for Arab women in Qatar to engage in breast cancer screening activities. We also inquired about what men participants perceive as facilitators and barriers to women’s breast cancer screening and what information would benefit the women and the men. Socio-demographic data was collected at the start of the interview using a socio-demographic questionnaire (Table 1).

**Table 1:** Selected demographic data of male participants in this qualitative study.

Characteristic	Categories of Data	Male (N = 50), N(%)
**Age (years) in average**	-	42± 10.7
**Nationality**	Qatari	13 (26%)
	Other Arabic	37 (74%)
**Years in Qatar in average**	-	22± 18.3
**Marital Status**	Single/never married	10 (20%)
	Married	40 (80%)
	Divorced	0 (0.0%)
	Separated	0 (0.0%)
	Widowed	0 (0.0%)
**Children**	Yes	39 (78%)
	No	11 (22%)
	Average number of children	4± 2.4
**Education**	Never went to school	2 (4%)
	Primary/junior high school	3 (6%)
	High school	8 (16%)
	Trade school	10 (20%)
	University Bachelor’s degree	24 (48%)
	Masters or PhD	1 (2%)
	Missing	2 (4%)
**Employment status**	Work full time	41 (82%)
	Work part time	2 (4%)
	Self-employed at home	0 (0.0%)
	Full time home-maker	0 (0.0%)
	Retired	1 (2%)
	Unemployed	6 (12%)
**Education level of spouse**	Never went to school	3 (6%)
	Primary/junior high school	4 (8%)
	High school	8 (16%)
	Trade school	5 (10%)
	University Bachelor’s degree	16 (32%)
	Masters or PhD	2 (4%)
	Missing	12 (24%)
**Religion**	Muslim	50 (100%)

### Data analysis

Data collection, analysis, and interpretation occurred simultaneously. NVivo 9, a qualitative data analysis software program was used to organize narrative data into themes and subthemes. In the early stage of data analysis, the transcripts were coded and a coding tree was be generated. These codes and the coding tree were refined as more data is collected and analyzed into emerging themes and subthemes. In detail, the data analysis steps involved (a) line-by-line, initial reading of the raw data to acquire a sense of the whole text; (b) being immersed in the data and extracting significant statements or recurrent patterns through interpretive reading; and (c) formulating and comparing meanings of each significant statement within and across data set transcripts and identifying recurrent patterns and categorizing them into clusters of themes [34]. The research team discussed the categorization of themes and subthemes. With 50 men participants, data saturation was obtained where discovered information was repeated and the phenomenon became stronger and more evident [35]. To minimize uncertainty and ambiguity in researchers’ interpretations, validation of the researchers’ reconstruction of meanings was performed through the second interview with participants. All of the ten male participants who volunteered for the second interview validated the preliminary interpretation of the data and commented that it reflected accurately their perspectives. The data gathered from the second interviews were incorporated into the categorized themes and the team examined the analyzed data for linkages to existing literature that best explained the findings [26].

## Findings

### Facilitators to participation in breast cancer screening

#### Breast cancer screening is important for women’s health

Of the 50 men interviewed, 41 indicated BCS examinations were important for a number of reasons. Prominently mentioned were the woman’s health and her central role in the family and in society.

Check-ups are vital not only for the well-being of the woman but also for the good of her family and society…Women are the base of society. The woman could be a mother, a wife, a sister, etc. One can’t do without her. The safer the family, the more stable it is. The whole family has to be well-rounded, aware, educated, healthy, etc. We really need to take this topic seriously…. So no matter what one’s gender is, one was given birth by a woman. It is the woman who takes care of the kids. She has to be in a good health so that everybody else in the family is in good health.

(Mohammed)

Early and frequent breast examinations were deemed necessary in order to detect breast cancer at an early stage.

It helps discovering diseases at their early stages and also curing them before it is too late.

(Ali)

It is very important to detect the disease in its early stages.

(Abdullah)

The breast cancer early detection examinations are always important and they contribute in the treatment success and in avoiding the dangers resulting from the cancer such as excising the breast [mastectomy] and the spread of cancer to other parts in the body and even death.

(Hassan)

It was generally perceived that early breast cancer detection led to a better prognosis of treatment.

It is important to me because I witnessed two cases. The first case was a death situation. The woman knew that she had Breast Cancer at its last stages; meanwhile, the second case was at its early stage and eventually was cured.

(Jabir)

Only three participants were unsure of the benefits and importance of screening activities. One indicated that they were not necessary ‘Every person is free. But for me, for my mother and sisters, it is not necessary to do them’ (Saad); one thought that screening should simply be included in regular checkups ‘the check-up must be general not specific’ (Carim); and one said that checkups were only necessary if one had symptoms ‘If my wife and I, both, witness the same symptoms, then I will encourage her to go through a check-up. Yet, if there isn’t anything, she should not bother consulting a doctor’ (Gadi). The rest of participants did not specifically discuss the necessity for screening activities although some mentioned regular examinations.

#### Arab men’s role in ensuring women partake in breast cancer screening activities

The participants felt that their role in supporting their wives was an important part in ensuring that breast examinations were done.

Men have an important role in the women’s life (father, brother or husband), they have to take care of her and take her to the hospital when it is necessary.

(Najib)

Our religion, Islam, asks us to look after women and take care of them. This shows how important and how valuable women are in Islam. Moreover, the woman is the foundation of the society; taking care of her means taking care of the whole society. She plays an important role in the success of each and every man. Therefore, it is a must that Arab men have to protect her and keep her away of everything that could harm her or impact her life.

(Yasser)

Most men interviewed advocated very strongly for breast examinations, indicating that they would encourage and support their wives in this matter. One participant not only promised to support his wife but to make sure that she participates in screening.

It's a must for me and any other person to support woman in all domains, especially in this subject because it is a very dangerous matter and it is a disease… It's a must to support her in this matter, because first of all it' s a matter of health.

(Fadi)

I encourage women to do the examinations and especially the self-examination.

(Elias)

I see and I encourage all women to do these examinations; frankly speaking, it is something very important and there is no shame about it.

(Yasir)

I will definitely encourage her and follow up with her, myself, in all cases.

(Nabeh)

Several participants felt that in general Arab women are reluctant to have their breasts examined by others, out of modesty or shame. They felt that in general Arab women are reluctant to have their breasts examined by others because breasts are viewed as sensitive private body parts. Women would feel very shy and embarrassed to uncover their bodies even to other females. Thus breast self examination was advocated mainly because women can do it themselves without the necessity of seeing/examining by a doctor.

Once the woman knows how to do self-screening, she will be always able to do it at home without bothering herself going to the hospital so often.

(Ali)

Overall, it is very encouraging to note that the majority of Arab men participants support BCS, recognize that such screening is important for women’s health, and they do not object to women having their breasts examined by health care professionals.

#### Knowledge of breast cancer screening activities

Most men had a basic knowledge of some or all breast screening activities (self-examination, clinical examination, mammogram) to detect breast cancer even though they might not use the proper terminology or had any detailed knowledge of how the screening is performed. Two men were very knowledgeable about BSE:

It could be through palpation, by hand; when touching her breast, the woman could feel something rigid or a tumor in the breast. She may perform this examination when lying down. To examine the right side of her breast, she palpates it with her left hand and vice versa. She should palpate the breast in a circular movement to detect any tumor, mass or change in the skin. When infected with breast cancer, the breast shape changes and the nipples’ shape and size change as well, the color of the breast changes, it becomes reddish … The woman should always take care of herself and look for any change in her breast. The woman should always when taking her shower and after the period, examine her breast looking for any problems and in the event she feels anything strange, she should immediately visit the physician.

(Elias)

You tell women: you stand in front of the mirror and you put your hand, because when she lifts her hand, she would feel her breast is tightened and it would be easier to feel any mass or change in the breast. Then, you should start to compress the breast, in the event there are any secretions with bad odors, this means that she has a problem in her breast. Third: lie down on the floor for example and compress the breast, if you feel any pain, this means that there is a problem in your breast.

(Mukhtar)

Although nine participants made no specific mention of BSE, the more typical comments were:

The check-up can be done at home. The woman feels around her breast to check if there are any tumors and whatnot.

(Munir)

Watching herself in the mirror is a habit and a nature of females. When there is something wrong, the woman notices it right away when she is in front of a mirror.

(Kareem)

The woman feels around her breast every once in a while … The woman has to use her hands to feel her breasts, while taking a shower, to know if there is anything abnormal or not.

(Gadi)

Although most participants were not very clear as to what the breast examination consists of, 38 participants mentioned that clinical breast examination is an examination performed by a physician.

The patient has to answer all the questions asked by the doctor with honesty to avoid any misunderstandings. Right after that comes the phase of analyzing her. The doctor will have to physically check on her breast, and then take a little bit of blood from her for blood analysis. So, a blood analysis and biopsy is one possibility. Another possibility is urine or stool analysis.

(Kareem)

Breast cancer check-up happens as the doctor takes out some cells from the breast and examined it in order to see if the cancer cells are active or passive.

(Sharif)

When a woman feels something wrong with her breast, she consults a doctor who checks on her and then suggests a check-up by radiologist.

(Talib)

The clinical examination was also perceived as protecting against not only breast cancer but other diseases as well. ‘*If the check-up is done regularly*, *people will avoid being infected by many diseases’* (Akbar). Although twelve participants made no mention of clinical breast examination, they made references at some point in the interview to ‘check-ups.’

Mammography was often referred to as x-rays, radiology, MRIs or imageries. Thirty five of 50 men described mammograms as a type of x-ray that detects abnormality in the breast and the other 15 did not mention anything about mammograms.

It may be radiations. That is possible. But it is not any radiations. Definitely, they have a name but I don’t know what they are called.

(Gadi)

I know that there is an X-ray imagery for the breast that is called…I forgot its name, but there are X-ray imagery examinations.

(Suhail)

One had good knowledge of what mammograms could do,

Last but not least, the third type of check-ups is the mammogram. That is the most perfect check-up known so far as it is very precise. It is the best technology out there up to now. Mammography allows the doctor to check on very microscopic substances that neither the woman nor the doctor can see. Such substances are usually located inside the breast and can’t be seen or felt by the doctor.

(Zakarria)

Three participants indicated that the suggested guideline for mammograms is once a year. Six men out of 50 said they didn’t know much about any type of breast examination even though they had heard about breast cancer.

#### Perceived knowledge of breast cancer screening guidelines

At present, the Qatar Supreme Council of Health recommends that women 45 and older should have breast cancer screening—mammogram every three years [36]. At the time of which this study was conducted (2011 and 2012), the Qatar guidelines recommend a monthly breast self-examination starting at age 20, a yearly clinical breast examination for women aged 35 and above, and an annual mammogram for women aged 40–69 years, unless otherwise advised by physicians. Several men knew details as to screening frequency guidelines in Qatar, the ages when breast cancer screening is appropriate, or where to go for clinical examinations and mammography. Predominantly, men referred to screening activities as examinations by doctors.

Of the men interviewed, 45 specifically indicated that breast screening activities should be performed on a regular basis, and many mentioned a check-up every 6 months or a year.

If the woman wants to protect herself, she has to go through a check-up at least once a year. Regarding the self-check-up, this could be done every now and then. It shall not be tied to a specific period of time. However, regarding the clinical check-up or the mammogram … it is supposed to be done at least once a year; it is preferable to be done once per six months.

(Amir)

Nineteen men perceived breast examination is important for older women (40 and up) and women after menopause. Some indicated younger women should do breast exam as well, but could just do BSE unless they find something abnormal.

Women after the menopause should do regular examinations because they are subject to this disease more than the others so focus should be made on old women more than young women. So old women should never neglect the examinations even if nothing is detected from the self-examination…. As for younger woman, 30-year old women may—if they are doing the self-examination—postpone the physician visit on condition that nothing is detected.

(Elias)

The symptoms of Breast Cancer appear after the age of forty. It is usually between forty and fifty.

(Nabeh)

Such examination should start from the age of puberty 12 or 13 years old.

(Omar)

Five men specifically acknowledged the existence of clinics in Qatar that specialized in women’s health and which employed female doctors.

There is a hospital in Hamad and in Al Khor called is specialized in women only, where the woman can go through regular check-ups.

(Faisal)

There is a medical center called “The Well-Woman’s Medical Center” where women can enjoy the privacy of being checked up by female doctors.

(Jamal)

We have to make people aware that there are female departments where doctors who conduct the check-ups and the whole staff are all females. That is something that ensures the security of the man.

(Zakarria)

The rest of the men seemed unaware or unsure that such facilities existed but thought that such clinics would be helpful in reducing the barriers for women to participate in BCS.

I advise that there be female medical center to sensitize and make people aware of that … There must be female specialists who have the ability of doing the breast cancer examinations.

(Khalid)

### Barriers to participation in breast cancer screening

There are a number of perceived barriers to breast screening activities which may contribute to explain why the rate of such activities is low amongst Arab women in Qatar. These are: gender related requirement that women should be examined by female doctors and the need for women to obtain approval by males for such examinations; the social taboo of talking about private body parts and sexual matters; the fear resulting from the social stigma of being diagnosed with breast cancer; and lack of awareness both of the disease itself and the signs and symptoms to look for. A few men mentioned financial barriers but primarily in relation to other Arab countries.

#### Gender related barriers

In many Arab societies, the man’s role is to protect, lead, and instruct female family members. Thus male’s perception and approval of BCS examinations are of significant important factor influencing women’s participation in breast cancer screening activities.

Almost all participants (45 of 50) emphasize that the gender of the doctor performing the clinical check-up/examinations was extremely important to them. Many of them repeated this opinion several times throughout the interview.

It is possible to teach the woman how to perform the self-examination and she can do it whenever she wants…. But to go and get examined by a [male] physician, no… she goes to the physician but she doesn’t let him examine her, she does the X-ray imagery.

(Saad)

It is not so urgent that the woman has to do the check-up right at the moment. If there is no female doctor available, she can wait. She will not die right away.

(Kamal)

Opinions on this topic range from one extreme, where everyone in the hospital or clinic should be female,

Another good factor of encouragement is that if there were hospitals only for females, this would help much more. If the staff is only females including doctors, nurses, receptionists, etc., this would also help a lot. As I told you, many men would not accept the fact that a male doctor touches the breasts of their wives. I am open-minded but still I won’t accept that a male doctor touches my wife’s private parts.

(Gadi)

through a slightly more moderate position.

I, as an Arab man, I won’t accept that my wife goes and does these examinations unless I am sure that the treating physician is a female.

(Bilal)

With an exception of one man who stated: *‘the gender of the doctor does not matter at all*’ (Kareem), eight of the 45 men indicated that they agreed for their wives being examined by a male doctor only in the case of life and death and when the woman has no other choices. All the rest strongly objected to clinical breast examinations being performed by a male doctor under normal check-up.

A woman would go to a male physician only when her disease is so developed and advanced and after making sure that there is no female physician available; when she has no other choice than death or going to a male physician, she would go to a male physician.

(Elias)

However, if needs be in urgent cases and where a female physician is not available, she would go only in urgent cases to a male physician. Otherwise no.

(Saad)

If it is a life-death matter, then there is no burden if it was a male doctor with a condition that he must be good and honest.

(Gadi)

In the Arab tradition, many women must get permission from their husbands or fathers in order to go for clinical examinations, and may be required to have male family member accompany them to their appointments. This can present as a significant barrier if the male family members are unaware of the importance of such check-ups and/or oppose breast examination.

She might have not finished things at home, taking care of the kids and other things make the husband not letting her go to the health centers.

(Daoud)

For those girls who are not married yet, the father will have no confidence to let his daughter to go and do such examination, because we are Arab and we are attached to [customs] and traditions. This matter is difficult to allow. I guess the main obstacle will be the parents and the husband if he has no idea about the subject or they don't know how important this [is].

(Omar)

Another reason that might prevent her of doing the check-ups is that she has to ask for permission from her husband or her parents.

(Kamal)

In the Arab world, Qatar for example, it is not appropriate that a woman consult a doctor by herself. She has to be accompanied by a man.

(Zakarria)

The Arab woman also needs somebody to accompany her to the hospital.

(Isam)

#### Social stigma and anticipated negative consequences of breast cancer

Social beliefs and values contribute to men’s perception of breast cancer and women’s level of participation in screening for breast cancer. The conceptualization of the body as private, of breast cancer as fatal, and the anticipated negative consequences of being diagnosed with the disease greatly influences the ways in which Arab men think about breast cancer and Arab women seek and receive examination for it. Fifteen participants indicated that even to talk about breast cancer in Arabic society is a very sensitive topic. Throughout the interviews, breast cancer was often referred to as “that disease.”

Her family members didn’t say it is cancer. They used to name it ‘that disease.’ They don’t like to speak about this disease and until now they don’t like to talk about it; people are usually disturbed of these issues and even of the name of the disease. And that’s why it is a problem to go and do the examinations or to say that someone is infected with this disease … It is shameful to ask about this disease and more specifically about women’s disease … People don’t like to name it, they call it: that disease. They don’t pronounce the name cancer. They say: such or such is infected with this disease without pronouncing its name.

(Saad)

In the Arab society, especially in Qatar, for a woman, talking about breast cancer in front of somebody is so hard; even in front of a woman like herself. She might feel shy and embarrassed. The cultural values and traditions prevent her from talking about such a disease.

(Talib)

Participants pointed out that in their culture, discussion of topics related to sexuality and private parts of the body is considered taboo. Typical comments were:

In the Islamic and Arab world, speaking about such topics that are more of sexual health issues is considered a taboo.

(Latif)

I believe that it is an embarrassing topic to speak about even among men.

(Gadi)

I have never talked to anybody about it. I thought that this is something only women can talk about.

(Faisal)

No, I have no idea about them and I don’t ask about them because if I do and I am a man, they would say: why are you interested in this issue?

(Saad)

I think that the only thing that hinders women from going and having a checkup is her shyness to undress in front of a man and the fear that a man might be staring at her breasts and we both know what might happen due to that.

(Abdullah)

The perception of social stigma if a woman is known to have gone for a clinical breast examination, is diagnosed with breast cancer, or has her breast removed, was mentioned by one-third of the men.

She may be afraid of the society’s perception; if a married or unmarried woman goes to do the examinations only for reassurance, there would be a rumor that this woman is infected with the disease even if she is in good state. An unmarried woman may be afraid that this rumor could influence her future that not one would marry her.

(Hassan)

Arab women are usually afraid of the view of the society towards them. She would think that if her friends knew that she went for breast cancer check-ups, they would start making up stories and as it might cause her problems with her suitor. If she is married, she thinks that her husband would divorce her, etc.

(Khalid)

From a social, religious and moral perspective, a woman would prefer to die than to be living with one breast only. One is susceptible to social embarrassment.

(Faisal)

Participants suggested that a major barrier to Arab women’s willingness to undergo breast examinations is an anticipation of negative consequences with family relationship if being diagnosed with the disease and breast removal. They perceived that it is common for a woman to be afraid that her husband might divorce her because she has breast cancer.

She will be afraid of the community’s perception of her. They would look at her and say she is poor, she is sick. People will feel pity for her because she is so sick and tired. Even more, she may be embarrassed and fear that her husband knows about her problem … She would be afraid if her family knows about her disease because this would cause a problem: her husband would get married again. So she would be afraid to say it or to go for the examinations… If she is young and infected with this disease and is obliged to excise it [mastectomy], how would her physical appearance be? Her husband would get married again and this is a great problem.

(Mukhtar)

The biggest fear is that discovering such a disease may lead to divorce. People know that cancer is a malignant and dangerous disease. That is the reason why the husband, as a result of self-protection, may end up divorcing her. And if she is still single, she would be afraid that she can’t get married when she discovers that she is infected.

(Rafi)

The perception that cancer is fatal or that it is contagious has greatly fuelled social stigma against breast cancer. A few men commented:

If a woman is infected with this disease, her life is ended… Rare are the women who are cured of breast cancer. Usually they suffer for three or four years and then they die.

(Saad)

When people know that one has cancer, they stay away from [her] and keep distance as they think that it is contagious… when the family members know that their mother or sister is infected by breast cancer, they would be afraid… The kids also would stay away from her.

(Ali)

If a woman is infected with breast cancer and is breastfeeding her baby at the same time, this could be dangerous for her baby and this disease could pass to her baby.

(Fadi)

#### Educational barriers

Some men (five participants) also perceived that the low level of education of some women contributed to a lack of awareness of breast cancer and the precautions one should take.

If they [women] are not educated and not aware they would not recognize the danger of this disease. Thus they would not accept to go and do the examinations. So when you speak with them about this issue, their reaction will be negative for sure.

(Yasir)

In the Arab world, many women have a limited educational level. An educated woman perceives this disease logically and can understand the efficiency of the early detection examinations and their influence on life.

(Hassan)

Our neighbor, Glory to God, is afraid of going to hospitals and of suffering from this disease, so if you tell her to go to the hospital, she won’t accept. She says that she has a tumor in her breast and she went to the hospital for the first time where they asked her to do examinations, but she refused. She is an old woman; and [she] didn‘t come back to the hospital. First of all, she is illiterate, she is not educated and she is old, she is around 50 or 60 year-old and she grew up in a rural region.

(Wasim)

When a woman is not knowledgeable and not aware of breast cancer, she would definitely ignore any symptom of breast cancer and not pay attention, thinking that it is just a minor issue.

(Latif)

Other women are denying the disease and saying that I am well, I am not sick, I am a live, thanks God that I am not feeling any abnormalities. May be she is the primary stage and she did not discover that.

(Najib)

#### Financial barriers

The cost of examinations and treatment was mentioned briefly by six participants. However, examinations and hospital care are essentially free to all residents of Qatar whether citizens or not, this reflects more on the potential cost in other countries in the region or on the lack of knowledge that such services are provided at no cost in Qatar.

In Egypt, check-ups are paying. …Financial reasons could be a strong preventing factor…. It is costly in Egypt and free in Qatar.

(Talib)

Some women don’t have money to go through a check-up because breast cancer requires a good amount of money.

(Yusef)

Mothers always sacrificing for their kids and don’t like to spend their money for unnecessary things.

(Najib)

Table 2 presents summary of the findings which include the percentage of male participants’ responses to questions that generated findings of this report.

**Table 2:** Summary of the responses of Arab men to the questionnaire on knowledge about breast cancer screening.

Questions	Most common replies	Male (N = 50), N (%)
**Perceived facilitators to participation in breast cancer screening**	Breast cancer screening is important for women’s health	41 (82%)
	Arab men’s role in ensuring women partake in breast cancer screening activities	45 (90%)
	Having knowledge of breast cancer screening activities	44 (88%)
	Perceived knowledge of breast cancer screening guidelines	45 (90%)
**Perceived barriers to participation in breast cancer screening**	Gender related barriers	45 (90%)
	Social stigma and anticipated negative consequences of breast cancer diagnosis	15 (30%)
	Educational barriers	5 (10%)
	Financial barriers	6 (12%)

### What the Arab men participants wanted to know

When participants were asked what information they wanted in order to help encourage the women in their families to participate in BCS, most indicated the need for details as to what were the causes and symptoms of breast cancer, and the methods of breast cancer screening examinations and treatments.

Prevention starts with knowing the causes of the disease. I would like to know about the examination methods and how it is performed to be able to talk with my wife about it and explain to her about it.

(Elias)

I like to know how these examinations are performed, who performs these examinations and where: in private or public hospitals—the examination methods and the treatment as well as some advice on how to avoid this disease or if there are some special behaviors and acts that could help prevent this disease.

(Yasir)

At the termination of the interviews, it was clear that many men not only wanted more information about breast cancer and screening activities, but were prepared to actively seek out such information for themselves. They were also very supportive of programs to increase awareness of the seriousness of the disease in Qatar and the promotion of breast cancer screening activities, both of these aimed not only at women but at men as well, in order for them to better protect and encourage their wives and daughters.

[Promotion should be] through the same awareness methods as women. Men have an effective role with this regard and he has a great effect whether for his woman or daughters. That’s why men should be aware of this disease and should be educated about how to deal with this disease if it is detected.

(Yasir)

The best way to do it is by attracting men’s attention to how dangerous this disease is. In the end, this woman could be a mother, a sister or a wife. So, it is a must to educate men about this topic, so that they educate women about it. As long as such disease infects the woman in a private and sensitive part of her body, there is nothing wrong in making men aware also of how hazardous breast cancer could be. This way, it would make more sense to a man that he encourages the females in the family to undergo the check-ups.

(Rafi)

Finally, simply by participating in the interviews, the men indicated that they were appreciative of the information about breast cancer and screening they received and that they gained the incentive to further their knowledge in order to better care for the women in their families.

The thing is that you drew my attention to this subject again. This interview made me more aware of my obligation towards women. You reminded me that I should talk to my parents about this and ask females in my family to do breast cancer check-ups. Thank you so much for that.

(Rafi)

What can be done to encourage women can be done to encourage men also.… A man has a mother, a sister, a wife, etc., so I don’t think that he would not care in case he was provided such information. By the way, you encouraged me to start doing research about this topic. I would like to obtain more knowledge about breast cancer. I would like to be an active individual in the society. One needs to know what to do in case confronted with such a disease. So, making both genders aware is so essential. This topic interests both genders.

(Yusef)

## Discussion and recommendations

In this study, we examine Arab men’s perceptions about breast cancer screening activities and their influence on the women’s health. It was found that there are both facilitators and barriers to women’s participation in breast cancer screening activities. As with many countries in the world, Arabs in Qatar characteristically are adherent to patriarchal customs and traditions. Both sexes believe that the role of men is to protect and support their wives and families. This encompasses financial support as well as protection of women's modesty [37, 38, 39]. Most men participants appreciated the importance of regular breast cancer screening in promoting women’s health and in early detection of breast cancer. They felt they had an important role in encouraging the women in their lives to participate in breast cancer screening activities, but were adamant that any examination must be performed by a female health care professional. Many participants strongly objected to having a male doctor perform clinical breast examinations unless it is under extraordinary circumstances where women would not have any other choices. This may be a non-issue in Qatar, where there are a number of clinics with female doctors who can be called on to examine women, and several men indicated that they were aware of their existence. Nevertheless, the fear that a woman’s breasts may be exposed and palpitated by a male health care professional can be a contributing factor in the reluctance of women to participate in such screening activities or in getting their husbands’ or fathers’ approval for breast examinations.

It is evident from the interviews that most Arab men are aware of breast cancer and they acknowledge the importance of early detection of this disease. However, while most men participants had a basic knowledge of the various screening activities such as self-examination, clinical examination and mammography, few participants knew details about the guidelines for screening in Qatar and the benefit of early detection regarding treatment and survival prognosis. Here there are implications for the need for more awareness about breast cancer among Arab women and the importance of discovering breast cancer in its early stages. Information needs to be disseminated that breast cancer in its early stages does not have symptoms and that breast cancer if treated in its early stages will have better prognosis and chance of survival for women. When a patient does experience symptoms, breast cancer is in the late stages. Most participants see their role as one of protector, supporter and educator but lack the knowledge necessary to carry out that role with any clear direction. Therefore, detailed information as to what breast cancer screening activities consist of, when such activities should take place and where, need to be disseminated to both women and men.

It is very encouraging to note that at the termination of the interview, many participants indicated an interest in learning more about breast cancer and screening activities in order to better help and inform their female family members. They were eager to learn and offer suggestions as to what information they need to extend their knowledge on the issue. Drawing on the findings of our interviews with male participants and others findings of this study in Qatar [16, 26, 40] and a comprehensive literature review of studies [41] we recommend a promotional campaign to provide public education about breast cancer and cancer screening methods, using language-appropriate and culturally sensitive educational material/ programs via variety of media means and campaigns to women and men (as gaining male support remains crucial for women’s health in Arab populations [40]. Initially, the campaign could last for at least a year, with the possibility of an extension if it proves effective. A promotional campaign to address knowledge gaps regarding breast cancer and its screening could include:

- A website, built in Arabic and English, which could include detailed, gender-appropriate information about breast cancer, screening activities, the revised Qatar guidelines, clinic locations, availability of female health care professionals, etc.
- Gender appropriate brochures with links to website, also with gender appropriate information and suggestions, for distribution in hospitals, pharmacies, doctors’ offices, universities, libraries, etc.
- Awareness posters on street billboards (very effective in Qatar) with link to web
- Information, workshops aimed at physicians and other health care workers about the need to recommend BCS to both female and male patients.
- Public, gender-separated lectures with prominent public figures on the need for BCS.
- Radio show, TV show, or commercials on both media with information on the need for and benefit of breast cancer early detection.
- Newspaper stories, advertisements with links to website.
- Use of public figures, influential women who either regularly participate in BCS or who recovered from BC because of BCS in billboards, ads, brochures, etc.
- Provide information to religious leaders, asking for their support, and working collaboratively with them to address women’s health issues.

Future research should investigate the effectiveness of public promotional campaign and additional factors that younger generations of women and men living in rapidly changing societies like Qatar might face, including the interplay of modernity, traditional and cultural practices, changing gendered roles and expectations, and increasingly higher income, employment and education levels among Arab women and men. More intervention and evaluation studies are also needed in this area to further develop culturally sensitive interventions and assess the cost-effectiveness and long-term sustainability of the programs.

### Limitation of the study

Owing to the nature of qualitative research, the findings of this study cannot be generalized as the perspective of all Arab men in Qatar. Furthermore, data collected from self-reported face-to-face in-depth interviews might be subject to recall or social-desirability response bias. According to Ganster and colleagues (1983), in an effort to conform to social norms, individual may give favorable responses which they perceive as acceptable by researchers and others regardless of their true feeling or actual behavior [42]. The social-desirability response bias may pose a threat to the rigor of research findings, thus researchers of this study made great effort to ensure participants that there are no right or wrong answers and that we were truly interested to know about their perspectives regarding breast cancer and breast cancer screening. Despite the study’s limitations, knowledge gained from this study give insights into how Arab women’s breast cancer screening practices can be supported by Arab men, which can be benefit to not only women in Qatar but also women with similar sociocultural backgrounds throughout the Middle East and globally.

## Conclusion

The incidence and mortality rates of breast cancer are rising in Qatar. Detection of breast cancer in late stages will also lead to a high percent of radical mastectomies and increased morbidity. If the theory—and evidence—based interventions developed is to create changes in breast health seeking behavior of Arab women, tailoring breast cancer screening interventions to the population’s unique needs and practices need to be taken [40]. Since Arab men’s opinions and support are major factors influencing their female family members’ participation in breast cancer screening, it is important that any program instituted to increase such participation be aimed at giving more information about breast cancer to not only Arab women, but also Arab men in Qatar. It is evident that Arab men are supportive of women’s health. To fully support Arab women’s breast health and to assist women to engage in breast cancer screening examination, more information is needed by the public as to the need for regular screening activities, the techniques used, the most current guidelines for breast cancer screening in the country, and the assurance of culturally appropriate and professionalism in all health care services. Given the expected doubling in breast cancer cases in the Middle East by 2030, a promotional program needs to be introduced in the near future in order to avert, at least partially, the social stigma, the social the high incidence and mortality rates of breast cancer cases in Qatar.

## Supporting information

S1 File Arabic men interview questions. “Arabic Men-Initial Interview Questions-May 20, 2017.docx”.
(DOCX)

S2 File Arab men narrative data. “Arab Men’s perceptions BCSE narrative data.docx”.
(DOCX)
