# Supplementary material for: Perceptions of Arab men regarding female breast cancer screening examinations—Findings from a Middle East study
Source: PLoS One. 2017 Jul 21;12(7):e0180696. doi: 10.1371/journal.pone.0180696 (PMC5521764; doi:10.1371/journal.pone.0180696)
Supplement: S1 File — “Arabic Men-Initial Interview Questions-May 20, 2017.docx”. (DOCX) [file pone.0180696.s001.docx]

**Study 2 – Qualitative Guiding Interview Questions**

**Factors Influencing Breast Cancer Screening Practices Amongst Arabic Women Living in the State of Qatar**

العوامل المؤثره في ممارسة الفحص الدوري لسرطان الثدي بين النساء العربيات في قطر

- • Fy Rʼyk ، Mādhā ʻLynā ʼN Nfʻl Ldʻm al-Mrʼh Llḩşwl ʻLá Āmtḩānāt Fḩş Srţān ath-Thdy؟
  • ʼY Nwʻ Mn al-Mʻlwmāt Ḩwl Srţān ath-Thdy ، Wʻrḑh ʼN Ykwn ʼKthr Fāʼidh Lkm Wghyrhā Mn an-Nsāʼ؟
  • Mā Hy ʼNsb Wʼfḑl Wsylh Bālnsbh Lnā Ltqdym Hdhh al-Mʻlwmāt؟
  • Kyf Ymknnā Tshjyʻ an-Nsāʼ ʻLá al-Ānkhrāţ Fy ʼNshţh al-Kshf ʻN Srţān ath-Thdy Mthl Fḩş ath-Thdy Khāşh Bhm ، Wālrʻāyh aş-Şḩyh Wālmhnyh Ydrs Şdwrhm ، Wālmāmwjrām؟
  • Wnḩn Nkhţţ Llqyām Mshrwʻ ʼĀkhr Lājrāʼ Bʻḑ at-Tghyyrāt al-Mnāsbh Ltshjyʻ an-Nsāʼ ʻLá al-Ānkhrāţ Fy al-Kshf ʻN Srţān ath-Thdy. Llqyām Bdhlk ، Nḩn Bḩājh Llʻml Mʻā Mʻ al-Jmyʻ Fy al-Mjtmʻāt al-Mḩlyh. Mā Hy ʼFḑl aţ-Ţrq Bālnsbh Lnā Llḩşwl ʻLá Dʻm al-Jmyʻ؟
  • Hl Hnāk Shyʼ ʼĀkhr Tryd ʼN Tkhbrny؟
- القاموس - [عرض القاموس المفصل](http://www.google.com.qa/dictionary?source=translation&hl=ar&q=&langpair=en|ar)

##### Initial Interview Questions for Arab Men

**Introduction**: *Thank you for taking the time to speak with me today. As you know, we are very interested in hearing what you think about how we can promote breast cancer early detection among Arabic women because we want to help to reduce breast cancer morbidity and mortality among women in Qatar. We will be discussing the sensitive issue of breast cancer and breast examinations for women, what you think might prevent or motivate women to have breast examinations, and what you think are the best ways of gaining support from Arabic men for the promotion of Arabic women’s breast cancer early detection. I want to assure you that all the information will be confidential. If there is a question which you don’t want to answer, all you have to say is “I don’t want to answer that question”. You also have the right not to participate in the project at any time. During our conversation, if you feel upset, we can stop the interview. We can also arrange for a health care professional to talk with you if you think that would be helpful.*

**أسئلة المقابلة الأولية للرجال العرب:**

**المقدمة** : شكراً لقضاء وقتك في الحديث معي اليوم. وكما تعلم ، نحن مهتمون جدا لسماع رأيك في كيفة تشجيع الكشف المبكرعن سرطان الثدي بين النساء العربيات لأننا نريد أن نساعد في الحد من الأصابه بسرطان الثدي وايضاَ الحد من الوفيات بين النساء في قطر. فنحن سنناقش مسألة حساسة حول فحص الثدي وسرطان الثدي للنساء، فما هو الذي يمنع أو يحفز النساء لعمل فحص الثدي ، وما هو في رأيك أفضل السبل لزيادة دعم الرجال العرب من أجل تعزيزالكشف المبكر لسرطان الثدي عند المرأة العربية. كما أريد أن أؤكد لكم أن جميع المعلومات ستكون سرية. وإذا كان هناك أي سؤال لا ترغب في الإجابه عليه ، كل ما عليك القيام به هو أن تقول "أنا لا أريد الإجابة على هذا السؤال". لديك أيضا الحق في عدم المشاركة في المشروع في أي وقت. أثناء حديثنا ، إذا كنت تشعر بالضيق ، يمنكك ان توقف المقابلة. ويمكننا أيضا ترتيب موعد مع اخصائين من الرعاية الصحية للتحدث إليك إذا كنت تعتقد أن من شأنها أن تكون مفيدة لك.

الاستماع

*First, I would like to start by finding out what you know about women’s breast cancer and breast cancer screening, and what you think prevents or encourages Arabic women to have breast examinations*.

أولاً ، أود أن أبدأ بما تعرفه عن سرطان الثدي النساء وفحص سرطان الثدي ، وما هو في رأيك يمنع أو يشجع المرأة العربية لعمل فحص الثدي.

***Prompt questions:***

- Please tell me what you know about breast cancer among women?
- Please tell me what you think causes breast cancer among women?
- Please tell me what you know about breast cancer screening examinations for women?
- What do you think /or feel about breast examinations for women?
- Do you think that Arabic women need to have breast examinations (breast self examination, clinical breast examination, mammogram) done regularly? If yes, why? If no, why?
- In your opinion, what would motivate women to have breast examinations?
- In your opinion, what would prevent women from having breast examinations?

**الأسئلة**:

- • من فضلك قل لي ما تعرفه عن سرطان الثدي بين النساء؟• من فضلك قل لي ما هي في رأيك أسباب سرطان الثدي بين النساء؟
  • من فضلك قل لي ما تعرفه عن فحص سرطان الثدي للنساء؟
  • ما هو رأيك / أو شعورك عن فحص الثدي للنساء؟
  • هل تعتقد أن المرأة العربية في حاجة الى القيام بفحص الثدي (الفحص الذاتي للثدي ، الفحص السريري الثدي ، والماموجرام) بشكل منتظم؟ إذا كانت الإجابة بنعم ، لماذا؟ واذا كانت الإجابة لا، ايضاَ لماذا؟
  • في رأيك ، ما الذي يحفزأو يشجع النساء على فحص الثدي؟
  • في رأيك ، ما الذي يمنع النساء من عمل فحص الثدي؟
- الاستماع

*Next, I would like to find out about what you think about breast cancer screening examinations.*

ثانياَ ، أود أن أتعرف على رأيك حول فحوصات الكشف عن سرطان الثدي.

- What effects your decision on whether or not you would support a woman to have breast cancer screening examinations? And why?
- In your opinion, what kind of beliefs and values influence Arabic women’s decision to have breast examination and mammogram?
- Would being a woman influence how an Arabic woman participates in breast cancer screening program? And why?
- What would be issues of concern for you and other Arabic men regarding women’s breast examinations?
- Why are you and other Arabic men concerned with these issues?

• ما الذي يؤثر على قرارك في دعم المرأة للقيام بفحوصات الكشف عن سرطان الثدي أم لا؟ ولماذا؟
• في رأيك ، ما هي المعتقدات والقيم التي تؤثر على قرار المرأة العربية للقيام بفحص وتصوير الثدي؟
• هل لكونها امرأه تأثير في كيفة امكانية المرأة العربية في المشاركه في برنامج فحص سرطان الثدي؟ ولماذا؟
• ماذا ستكون القضايا ذات الاهتمام لديك ولدى غيرك من الرجال العرب بشأن فحص الثدي للمرأة؟
• لماذا أنت والرجال العرب الاّخريين مهتمين بهذه القضايا؟الاستماع

• Mā Yʼuthr ʻLá al-Qrār al-Khāş ʼM Lā Ymknk ʼN Dʻm al-Mrʼh Lfḩwşāt al-Kshf ʻN Srţān ath-Thdy؟
• Fy Rʼyk ، Mā Hw Nwʻ Mn al-Mʻtqdāt Wālqym an-Nfwdh al-ʻRby
            Qrār al-Mrʼh Lfḩş ath-Thdy Wtşwyr ath-Thdy؟
• Fy Rʼyk ، Mā Mn Shʼnh Jʻl al-Mrʼh al-ʻRbyh adh-Dhhāb ʼW ʻDm adh-Dhhāb Llfḩş ath-Thdy Wtşwyr ath-Thdy؟
• Hl Yjry Tʼthyr al-Mrʼh Kyf Ymkn Lāmrʼh ʻRbyh Tshārk Fy Brnāmj Fḩş Srţān ath-Thdy؟ Wlmādhā؟
• Mādhā Stkwn al-Qḑāyā Dhāt al-Āhtmām Ldyk Wldá ar-Rjāl al-ʻRb al-ʼKhrá al-Mtʻlqh Fḩş ath-Thdy Llmrʼh؟
• Lmādhā ʼNt Wālrjāl al-ʻRbyh al-ʼKhrá al-Mʻnyh Bhdhh al-Qḑāyā؟

القاموس - [عرض القاموس المفصل](http://www.google.com.qa/dictionary?source=translation&hl=ar&q=&langpair=en|ar)

*I am also very interested to find out about what you think is needed in terms of breast cancer screening.* (Finding out about what participants perceive as the best possible strategies to promote breast cancer screening)

كما أنني مهتم جداً لمعرفة رأيك حول اللازم للكشف عن سرطان الثدي. (لإيضاح ما تعتبره المشاركون أفضل الاستراتيجيات الممكنة لتعزيز فحص سرطان الثدي).

***Prompt questions:***

- In your opinion, what should we do to support women in Qatar to fight breast cancer?
- What kind of information about breast cancer and its screening would be most helpful to you and other Arabic men?
- What would be the most appropriate and the best way for us to give this information?
- How can we encourage Arabic women to engage in breast cancer screening activities such as examine their own breasts, have health care professionals examine their breasts, and mammograms?
- How can we encourage Arabic men to support their female family members’ breasts examinations?
- We are planning to do another project to make some appropriate changes to encourage women to engage in breast cancer screening. To do that, we need to work together with everyone in the communities. What would be the best ways for us to get everyone’s support?
- Is there anything else you want to tell me?

**الأسئلة:**

• في رأيك ، ماذا علينا أن نفعله لدعم المرأة في قطر لمحاربة سرطان الثدي؟
• أي نوع من المعلومات حول سرطان الثدي ، والفحوصات الخاصه به يمكنها ان تكون أكثر فائدة لك ولغيرك من الرجال العرب؟
• ما هي أنسب وأفضل وسيلة لتقديم هذه المعلومات؟
• كيف يمكننا تشجيع النساء العربيات على ممارسة أنشطة الكشف عن سرطان الثدي مثل الفحص الذاتي للثدي، فحص الثدي من قبل اخصائين في الرعاية الصحية، وأشعة الثدي (ماموجرام)؟
• كيف نشجع الرجال العرب لدعم أفراد أسرهم الإناث لفحص الثدي ؟
• نحن نخطط للقيام بمشروع آخر لإجراء بعض التغييرات المناسبة لتشجيع النساء على الإنخراط في الكشف عن سرطان الثدي. للقيام بذلك ، نحن بحاجة للتعاون مع الجميع في المجتمع. فما هي أفضل الطرق للحصول على الدعم من الجميع؟
• هل هناك اي شيء آخر تريد أن تخبرني عنه؟

- الاستماع
