# Supplementary material for: Perceptions of Arab men regarding female breast cancer screening examinations—Findings from a Middle East study
Source: PLoS One. 2017 Jul 21;12(7):e0180696. doi: 10.1371/journal.pone.0180696 (PMC5521764; doi:10.1371/journal.pone.0180696)
Supplement: S2 File — “Arab Men’s perceptions BCSE narrative data.docx”. (DOCX) [file pone.0180696.s002.docx]

Arab Men’s Perception regarding Breast Cancer Screening Examinations – Findings from a Middle East Study

# Question: Interviewer: what do you know about the breast cancer screening examinations (BCSE)?

# Interviews separated into categories

## [METHODS OF BCSE – SELF EXAMINATION Page 2](#Cat1)

## [METHODS OF BCSE – CLINICAL EXAMINATION Page 10](#Cat2)

## [METHODS OF BCSE – MAMMOGRAMS Page 17](#Cat3)

## [NECESSITY OF BCSE Page 23](#Cat4)

## [REGULARITY OF PERFORMING BCSE Page 30](#Cat5)

## [AGE FACTOR Page 38](#Cat6)

## METHODS OF BCSE – SELF EXAMINATION

## The following 35 interviewees indicated knowledge of self-screening and/or techniques.

## Interviewee (100042)

You mean when the woman examines her breast. It could be through palpation, by hand; when touching her breast, the woman could feel something rigid or a tumor in the breast. She may perform this examination when lying down. To examine the right side of her breast, she palpates it with her left hand and vice versa. She should palpate the breast in a circular movement to detect any tumor, mass or change in the skin. When infected with breast cancer, the breast shape changes and the nipples’ shape and size change as well, the color of the breast changes, it becomes reddish

mainly the breast cancer self-examination, the woman should always take care of herself and look for any change in her breast. The woman should always when taking her shower and after the period, examine her breast looking for any problems and in the event she feels anything strange, she should immediately visit the physician.

## Interviewee (100088_n)

yes, yes always, women should do the home test periodically for it is very important to detect the disease in its early stages and thus would be cured by God’s will.

## Interviewee (200014_n)

Yes, because the self-examination, they may feel, just by touching , which means that the self-examination does not give a convincing indication, but at the hospital and with the specialists, they know better, i mean they are aware of this

## Interviewee (200087)

for example, for the self-examinations, it has many forms and types that could substitute the necessity to go to hospitals. Many women say that they don’t like to do the examinations or to go to the hospital; so if a woman is at home, she could stand in front of a mirror and examine her breast looking for any change in the breast size or color, any change in her breast. ...If a woman does the self-examination at home, she may notice a change in the shape but may not be sure if she has cancer or not; thus the X-ray imagery is a god means to be sure.

## Interviewee (6000149)

examinations? It is the X-ray imagery and the woman could examine her breast at home. So in the vent she notices any masses or something strange, she should immediately go to the physician.

**Interviewer:** so you mean that the woman should examine her own breast

yes, I watched a program where they said that the woman could examine her own breast first and in the event she notices any mass that is more rigid than the surrounding area, she should go to a physician.

## Interviewee (100019_n)

Sure I encourage women to do examinations not just for the breast but for all the body to be sure that she don’t have any problem and to learn to do self-examination.

## Interviewee (100020)

**Interviewer:** this examination or other examinations you don’t know which are: the self-examination performed by the woman herself

how is that?

in my opinion, women go to check and examine their breasts, if something is detected may God forbid, they go to private clinics specialized for cancer

## Interviewee (100050)

as I learnt from media and a campaign on the TV, in the event the woman notices anything that is abnormal like a mass or something like that, it is better to go immediately to a physician but I don’t know what the medical procedures are exactly; I guess it is about radiology

## Interviewee (100093)

I know that the examination is mainly performed by the woman herself; in the event she feels something in the breast by palpation, this should push her to do the examinations. If she feels something in her breast she should go and do the examination, especially if it is a mass in the breast

## Interviewee (100097)

**Interviewer:** do you know what these examinations are exactly?

no, I have no idea about them and I don’t ask about them because if I do and I am a man, they would say: why are you interested in this issue?

yes, I told you at the beginning of our conversation that our neighbor used to do the self-examination because I used to hear them saying that she had masses under the armpit and that’s how she knew that she had cancer.

## Interviewee (100098)

I know the types of examinations: the first one is the self-examination

the woman performs this examination herself.

## Interviewee (100099)

there is a self-examination that could be done.

a woman performs her breast personally and examines her breast nature; she looks for any rigidity, tumor or anything like that

## Interviewee (1000100_n)

the woman may detect that there are some changes in her breast. After the age of 40, in the event a tumor appears in the breast or between the breast and the armpit, or any swelling or change, this is considered a sign of disease or this would show that something is infecting her breast. She may feel these things and says that this is normal. For women, these things may appear after the age or 40. But before the age of 40 or before marriage, this also means that the disease is infecting her early.

the woman if she is afraid to go to the physicians or centers, she could compresses her breast with her fingertips, if she feels any masses or any secretions from the nipple with a bad odor or any mass around the breast or the skin of the breast is rough, those are all indications that the women may have cancer. In these cases, woman should immediately go to see the physician or do the necessary examinations to detect the stage of the disease. This is what I can tell you

You tell women: you stand in front of the mirror and you put your hand, because when she lifts her hand, she would feel her breast is tightened and it would be easier to feel any mass or change in the breast. Then, you should start to compress the breast, in the event there are any secretions with bad odors, this means that she has a problem in her breast. Third: lie down on the floor for example and compress the breast, if you feel any pain, this means that there is a problem in your breast. This ne of the steps

## Interviewee (100081)

The examination which we can call it or the woman call it for herself to see the shape of the breast by touching it, to see if there is any tumor in the body before going to the doctor

## Interviewee (600075_n)

As I heard, there are many examinations for this matter. There is a self-examination which the girl or the woman or the wife can do it by herself .... Concerning the self-examination, any girl, woman or wife can do it at home by standing in front of the mirror and check the size of each breast if there is any differences, it can be a proof that she is affected by the disease and it's better for her to go to the doctor. There is another examination while bathing to check, for example the right breast she raise up the right arm to her head and with the left hand she check the breast specially the part from the nipple the under arm, if she found any abnormality or any lump it's better to go to the clinic and there is another examination by lying on the floor, either she can check by the eye if no matching between the breast or she put a cushion under her left shoulder and check by the right hand the breast in the left. These are ways where the girl can do it at home and if she find any abnormality in the breast she can go tho the clinic to see the doctor. What are the signs the woman or the girl can notice if she did the self-examination, is the change in the color of the skin of the breast or lumps in the nipple or discharges or liquid or blood come out of the nipple.

## Interviewee (200084)

I don’t have any idea how check-ups are undergone. But I think it is the check-up of a specific area as tumors show up at the level of the breast. This could be known through the shape and the smoothness of the breasts, something like that [laughs.] This means that the woman has to check the smoothness of her breast but sometimes, she can’t find anything as the tumors could be either internal or external. Therefore, it is up to specialists to find out.

As long as it is a tumor, it must be something different appearing on the surface of the breast. That is most of the time clear as the woman can notice the difference between the two parts. She can tell that there is something unusual and additional.

**Interviewer:**  You have just mentioned the ordinary check-up that is self-checking the breast. Can you tell me more about that?

Hmm! I don’t really know much about that, actually. That was just a mere idea and I am not sure. However, reasonably, it is possible to sense the breast according to some specific criteria. For example, the woman can compare both breasts through the shape and she can, most of the time, figure out the difference between the right and left breasts as tumors usually appear around the breasts. Therefore, it is possible to sense through touching.

## Interviewee (200013)

It is a check-up that the woman does by feeling her breasts. She shall doubt in case she has felt any changes at the level of her breasts. We do believe that if a woman goes through a regular check-up, she will never be in a situation of breast cancer infection.

## Interviewee (200015)

What I know is that check-ups are two types. It could be a periodic check-up and it could also be through x-rays, MRIs and other radiology technologies. Periodic check-ups are usually done with the hands to check if there are any tumors or change in shape and size. In case of doubt, the case gets escalated to more special check-ups.

## Interviewee (200085_n)

People can simply go to hospitals to check with specialized nurses in order to show them how breast self-screening can be done.

Once the woman knows how to do self-screening, she will be always able to do it at home without bothering herself going to the hospital so often.

## Interviewee (200086)

As far as I know, Breast Cancer goes through many check-ups. The first check-up is feeling the breasts, watching it, observing the size of it as it may change in case of infection, and checking if there are any secretions.

## Interviewee (600018)

Actually, while at home, the woman can do self-screening. She has to consult a doctor immediately if the shape of the breast is different, especially the area around the nipple, or if the breast produces liquids

## Interviewee (600074)

Supposedly, for precaution reasons, women must go through screening whether by 1) Self-screening: standing in front of a mirror and doing the check-up

## Interviewee (600082)

Self-screenings have to be applied after each menstruation.

one of the symptoms of it is that the tumor causes pains and inflammations on the skin around the breast especially at the area of the nipple; the shape of the nipple changes. And in some cases, the nipples start discharging some liquids.

Well, this means that the woman can check herself up. So to say, after the menstruation, she can do the check-up by herself to know if there is anything wrong with her breast. She can know that through the changes that appear on the breast at the level of the skin and the shape; i.e., the size of the breast.

## Interviewee (600089)

Ah yes! I heard that in female hospitals, there are courses that teach women how to effectively check on the breast. There is a specific methodology that women use to check on their breasts after each course. The check-up is usually under the armpit and around the breast. When the woman feels anything abnormal, she has to consult a doctor as soon as possible.

Now, there is something in hospitals called “The Well Woman Clinic” where they are teaching women how to do self-breast-screening. That is a positive idea. Women will be able to tell if they have the symptoms or not at early stages.

## Interviewee (2000153)

So, as I said before, there are steps that women have to follow, which are the self-screening, clinical check-up and mammogram.

## Interviewee (30004)

The woman checks on herself and if she observes something, she must consult a doctor who knows better than her.

## Interviewee (30006)

Well, watching herself in the mirror is a habit and a nature of females. When there is something wrong, the woman notices it right away when she is in front of a mirror. The woman notices that there is something abnormal going on when she feels pain in her breast or on top of her belly. So, in such case, it is a must that the woman consults a doctor to get to know what she is going through. More pain could be felt, for instance, when the woman is breast feeding her baby. This could be one reason that obliges the woman to think about visiting a doctor.

## Interviewee (300051)

Well, first, the woman feels that there is a tumor or a substance around her breasts.

## Interviewee (300066)

Well, we consulted a doctor and I explained to him that we noticed something that was a little bit bumpy at the level of her breast. I mentioned that it had appeared only three or four days before we consulted him.

## Interviewee (800092)

I have heard that people are encouraged to go through check-ups. What I also heard is that the woman feels around her breast every once in a while. She is supposed to go through a consultation with a doctor in case she has felt something. The woman has to use her hands to feel her breasts, while taking a shower, to know if there is anything abnormal or not. However, though, I know that a check-up can be self-done.

## Interviewee (3000101)

As I said the woman still can go through a self-check-up by touching her breasts in order to notice if there are any breast cancer symptoms.

If it is before consulting a doctor, the woman usually stands in front of a mirror to check her breasts by touching around them.

## Interviewee (6000132)

What I know is that the woman stands in front of the mirror, holds her arms up, and then start checking around the breasts and under the armpits. When she notices any abnormality, she then needs to consult the doctor as soon as possible to have her breasts examined. That is all I know about the check-up.

## Interviewee (6000134)

What I know is that the check-up can be done at home. The woman feels around her breast to check if there are any tumors and whatnot.

## Interviewee (6000135)

The first type is the monthly self-screening of the breast where the woman periodically checks on her breasts by herself by feeling, touching and observing the area around the breasts and the armpits. Such a check-up is supposed to be done once the female reaches the age of twenty.

## Interviewee (6000136)

As far as I know there are more than one way to do Breast Cancer check-up. Such check-ups don’t necessary have to be regular as Cancer may happen anytime without symptoms. So, what I know is that women can do a self-check-up at home without a need to consult a doctor. Though, this check-up is not reliable as not all women are aware of how to do it.... This does not mean that women have to do without the monthly self-check-up. When a woman finds out anything unusual around her breast, she has to go to specialized center for a deeper check-up.

## The following 6 interviewees indicated no knowledge of self-screening.

## Interviewee (6000151)

No reported knowledge

## Interviewee (600077)

I have heard about check-ups but I don’t really know what they are.

## Interviewee (600083)

**Interviewer:** Actually, there are some special Breast cancer check-ups. Have you heard about them?

I heard but didn’t see. I didn’t practice and didn’t meet somebody practicing!

I heard that there are check-ups and there are also treatments.

## Interviewee (6000146)

Well, I heard about breast cancer check-ups but I have never experienced them.

## Interviewee (6000148)

No, I have never heard about them. Sometimes I watch health programs but not always; and I like to follow them.

## Interviewee (10005_n)

Breast cancer screening! No, no idea!

**Interviewer:** You have never heard about it?

No, never

## The following 9 interviewees made no mention of self-screening.

## Interviewee (6000147)

## Interviewee (6000150_n)

## Interviewee (600076)

## Interviewee (600078)

## Interviewee (600079)

## Interviewee (600080)

## Interviewee (2000154)

## Interviewee (6000131)

## Interviewee (6000133)

## METHODS OF BCSE – CLINICAL EXAMINATION

## The following 38 interviewees made either specific or general reference to clinical (medical) checkups as separate from mammography examinations.

## Interviewee (100042)

he will examine her and reassure her; if she doubts about anything in her breast or if she notices any change even without pain, it is better for her to see a physician because, treatments are always more useful when undertaken early. .... This is better than leaving the disease until it develops and then undergo the treatment

## Interviewee (100088_n)

I advise all women to periodically check themselves in the medical center.

## Interviewee (200014_n)

but at the hospital and with the specialists, they know better, i mean they are aware of this

## Interviewee (200087)

Some hospitals perform the cells tests, by taking samples from the cells or the blood to test them like the urine test for example to directly show if the woman is infected with breast cancer.

## Interviewee (6000147)

The breast cancer examinations may be performed as well through the biopsy – they take a smear from the breast and we examine it in the laboratory.

## Interviewee (6000149)

there are maybe other reexaminations performed in laboratories, but I don’t know exactly. Maybe they take a smear or they do an X-ray

## Interviewee (6000150_n)

I heard that there are annual examinations; I don’t know here, if at Hamad or elsewhere, but they are performing some programs and facilitation activities for women to get information and do the examinations

## Interviewee (100020)

there are as well the chemical tests through blood test

## Interviewee (100093)

She can do tests, blood tests and X-ray imagery in addition to the biopsy

## Interviewee (100097)

However, if she knows that she has cancer, she goes to the physician but she doesn’t let him examine her, she does the X-ray imagery.

## Interviewee (100098)

The second examination is the clinical examination performed by one of the medical body like a physician or nurse

## Interviewee (1000100_n)

Thus, the woman should immediately go to the specialist physician to detect the disease since its early stages and to avoid any side effects or other problems. This is about the disease discovery by the woman herself. If she feels any changes in her breast or any pain, she should immediately have recourse to the physician who should do a diagnosis through imageries or direct examination of the breast to detect the problem.

## Interviewee (100081)

or if there are any strange discharges in the body and she can see the doctor and after that she go for the examination for a regular examination and after 2 or 3 months she can see the doctor again

## Interviewee (600075_n)

I also read from the Net that there is a new device called Gill are used to determine the stage of the disease the person reach it and its danger.

there is also other things in the examinations like the screening of the body it shows calcium deposits as I heard the calcium deposits is a main cause of the breast cancers and so on.

## Interviewee (200084)

It is not so urgent that the woman has to do the check-up right at the moment. If there is no female doctor available, she can wait. She will not die right away.

## Interviewee (200013)

Breast cancer check-up happens as the doctor takes out some cells from the breast and applies check-up on them in order to see if the cancer cells are active or passive.

## Interviewee (200085_n)

Frankly speaking, I don’t know much about Breast Cancer because it is most of the time done in hospitals. It is a specific procedure done by the nurse in hospitals, that is touching and feeling some areas in the breast.

## Interviewee (600018)

Check-ups can be done by radiology or blood analyses. .... The doctor does the check-up either via radiology or analyses.

## Interviewee (600074)

2) checking with a doctor, preferably a female doctor in case they are embarrassed

## Interviewee (600076)

I know that people go to hospitals to do early examinations. However, I don’t know whether these examinations are on breast cancer or some other disease.

## Interviewee (600078)

Moreover, still, the doctor usually checks if there is anything abnormal regarding the color of the breast, the shape and the size of it. The doctor checks on the breast visually and by feeling it.

## Interviewee (600080)

It is like any other check-up. It is as if you are going to check-up on your eye or ear. It is a normal check-up. It is a good thing. When you check up, you take better care of yourself. It is good!

The second type is by simply feeling the breast. By touching, the doctor can determine whether there is a cancer or not in that it gets clear to him/her as the shape, size and smoothness may change and differ.

## Interviewee (600089)

The specialist uses the new invented equipment that is specialized only in breast screening and examination alongside with radiology and so forth.

## Interviewee (2000153)

So, as I said before, there are steps that women have to follow, which are the self-screening, clinical check-up and mammogram.

## Interviewee (2000154)

*Note: While filename 200154, interviewee was actually 6000154)*

One has to go through a check-up in order to see if she is infected or not at an early stage so that one can get cured easily.

I know that there are check-ups but I don’t know how they are undertaken. I think they take samples and they analyze them to check if there is any abnormal growth of the cells. As you know, the cells of the body keep changing. Some cells die and some other take over their places. If there is an abnormal growth of cells at the area of the breast, that is an indicator. Breast Cancer happens when the lymphatic cells get damaged by the cancer. This is related to the breast feeding cells inside the breast. I don’t know what the check-ups are frankly speaking but I know that there are things that can be done to avoid this disease.

## Interviewee (10005_n)

Well, the thing is that the woman consults a doctor when she feels something wrong at the level of her breasts. That could be an inflammation or a swelling; and these signs are not necessarily breast cancer symptoms. When the woman feels a tumefaction, she shall go to the doctor who, in turn, checks on the breast either by mammogram or anything else. I don’t know how the doctor conducts the check-up in fact.

## Interviewee (30004)

I don’t have any idea about that. I have never thought about how check-up are undergone. However, though, I think that the woman goes through a blood analysis. The doctors also checks on her.

Well, before using machinery, the doctor of course uses his hand to feel the breast and check if there are any symptoms to this disease. I don’t really know how it exactly goes but I know there are clinical check-ups that are used without medical equipment.

Well, before anything, the doctor checks with his eyes to observe if there is any change in the size and color of the breasts. That is what we call the symptoms to the disease. The next step is that the doctor uses his hand to feel the breast and check if there are any extra substances around the area of the breast. It is necessary that the doctor uses both, his hand and eyes. It is like any other disease. The doctor can’t recognize the disease from a distance. Next comes the machinery that is the radiology to make sure there is a cancer or no.

## Interviewee (30006)

It is done through blood analyses. The Woman needs to have knowledge about this tumor so that she consults a doctor once she notices its symptoms even though they are minor and not of importance. It is better to make sure she knows about the symptoms

When the doctor receives the patient, they start by asking both open-closed questions and closed-open question to extract as many details from the patient as possible. The doctor knows exactly what questions to ask. Examples of questions that could be asked during the consultation are what do you feel when you touch your breast? Do you feel any pain at your breast area? Do you have a baby?

Right after that comes the phase of analyzing her. The doctor will have to physically check on her breast, and then take a little bit of blood from her for blood analysis reason. So, a blood analysis and biopsy is one possibility. Another possibility is urine or stool analysis.

## Interviewee (300051)

Anyways, what happens is that when a woman feels something wrong with her breast, she consults a doctor who checks on her and then suggests a check-up by radiology.

## Interviewee (300066)

After we have explained to him how that thing felt, he, right away, started to check her breast using his hand to observe if there was anything anomalous.

## Interviewee (800092)

If any substance is felt, she has to go through a check-up. I know that women are always encouraged to go through such check-ups. What are they? I have no idea! I would say that she has to consult a doctor in case she feels any temporary pain in her breast. We are men, we don’t know much about that.

Then it could be a blood diagnosis.

The doctor will check on her breasts! He will have to feel them.

## Interviewee (3000101)

Then, she consults a doctor in case she has felt any change.

## Interviewee (6000131)

As far as I know, the check-up is undergone on the area of the breast in order to get evidences.

## Interviewee (6000132)

When she notices any abnormality, she then needs to consult the doctor as soon as possible to have her breasts examined. That is all I know about the check-up.

## Interviewee (6000133)

Well, one should go through a regular check-up every now and then without necessarily feeling any abnormality.

## Interviewee (6000134)

In the case of the presence of such tumors, she, then, shall consult a doctor. Other than that, I don’t know much about it.

## Interviewee (6000135)

The second type of the check-up is called Clinical Check-up. It is the phase where the doctor checks on the woman’s breast to observe whether there are any extra substances on the breasts area. What he looks at is the shape, the color of the skin, the weight, etc. In other words, the doctor uses his middle fingers to carefully check on any abnormality at the level of the breasts.

## Interviewee (6000136)

I also know that there is a clinical check-up, which should be done at hospitals. As to what my wife told me, the (female) doctor checks on the size and the shape of the breast.

## The following 12 interviewees made no mention of clinical examinations although later in the interview they made reference to “check-ups”.

## Interviewee (100050)

Not directly mentioned

## Interviewee (6000151)

Not directly mentioned

## Interviewee (100019_n)

Not directly mentioned

## Interviewee (100099)

Not directly mentioned

## Interviewee (200015)

Not specifically mentioned

## Interviewee (200086)

Not specifically mentioned

## Interviewee (600077)

Not specifically mentioned

## Interviewee (600079)

Not specifically mentioned

## Interviewee (600082)

Not specifically mentioned

## Interviewee (600083)

Not specifically mentioned

## Interviewee (6000146)

Not specifically mentioned

## Interviewee (6000148)

Not specifically mentioned

## METHODS OF BCSE – MAMMOGRAMS

## The following 35 interviewees made some reference to mammography, radiology, x-ray examination, etc.

Note: In one case, only the interviewer mentioned mammography while the interviewee indicated he had not heard of it previously (highlighted in yellow)

## Interviewee (100042)

And if she doubts about anything, she should go to the physician to examine her or do the necessary imageries; this is better even if she has nothing, but she could be reassured.

## Interviewee (100088_n)

the breast cancer test is usually done through the x-ray test; the symptoms however are swollen breasts and unnatural secretions of the breasts. The doctor would check the x-ray scan picture of the breasts and then diagnose the case.

## Interviewee (200014_n)

**Interviewer:** Have you heard about mammogram before?

Interviewee: No this is the first time

## Interviewee (200087)

Although she can do this examination at home, it is better for her to go to the hospital because there are many things there that could help in this examination better than performing this personally. For the X-ray for example, a woman could go to a nurse or physician and could do the X-ray imagery that detects everything or the cancer if any may God forbid.

## Interviewee (6000147)

yes. I know that there is an X-ray imagery for the breast that is called…I forgot its name, but there are X-ray imagery examinations.

## Interviewee (6000149)

It is the X-ray imagery

## Interviewee (100020)

as I think, the woman does the X-ray imagery for her breast to see if there is any strange body in her breast

## Interviewee (100050)

if the woman notices anything abnormal in her breast, she should do the examinations. I guess that there is a radiology

## Interviewee (100093)

There are examinations such as the mammogram that a woman should do at least once per year.

## Interviewee (100097)

However, if she knows that she has cancer, she goes to the physician but she doesn’t let him examine her, she does the X-ray imagery.

## Interviewee (100098)

and there is also the X-ray imagery to prove the presence of tumor

**Interviewer:** do you know what the breast X-ray imagery is called?

IRM

## Interviewee (100099)

There is also an X-ray examination. Those are my information. Medicine is very developed. Of course there are a lot of examinations, tests and imageries that could be done to reach cold results.

**Interviewer:** and what about the X-ray? Do you know what the X-ray is exactly?

no, I don’t know its exact nomination

## Interviewee (1000100_n)

the next step is the diagnosis through imagery, so you are going to do the imagery, don’t worry, it is a normal imagery, it has no side effects and causes no problems

## Interviewee (100081)

And also there is also the X-Rays.

## Interviewee (600075_n)

there is X-Ray in the clinics like the *** X-Ray or a new device called the mammogram.

Also when she visits the doctor they can use the mammogram, the *** X-Ray,

I only heard about it from one of the friends, and he told me that this is a tech used as a C.T.S. sort of X-Ray using computer for the examination and comparing, but I have a limited information about it.

## Interviewee (200084)

Now, technology had developed everywhere, so the check-ups could be undergone through X-rays.

## Interviewee (200015)

What I know is that check-ups are two types. It could be a periodic check-up and it could also be through x-rays, MRIs and other radiology technologies.

## Interviewee (200086)

There is also another check-up that there is done through X-ray radiations. That is to the best of my knowledge. I don’t have much knowledge about how this is done. So, it is X-rays and scans, that it would be able to do the exact check-ups on the cells

## Interviewee (600018)

Check-ups can be done by radiology or blood analyses.

## Interviewee (600074)

3) mammography check-up.

## Interviewee (600078)

I don’t know much about it, in fact. However, what I see in the hospital where I work, in Al Khor, is that there are specific departments where the check-up is done by radiology.

## Interviewee (600080)

What I know is that there are two types of examinations: The first one is that women go through radiology. It is when they get inside such a machine that scans the whole body.

## Interviewee (600082)

radiology and analyses must take place to diagnose it. I have here three types of radiology: X-rays, MRI and Thyroid scans.

## Interviewee (600089)

Yes, as I said before, the check-up can be done through radiology and mammogram

## Interviewee (2000153)

So, as I said before, there are steps that women have to follow, which are the self-screening, clinical check-up and mammogram.

## Interviewee (10005_n)

she shall go to the doctor who, in turn, checks on the breast either by mammogram or anything else. I don’t know how the doctor conducts the check-up in fact.

## Interviewee (30004)

That is done through radiology. The picture that I have in mind about breast cancer is that it is a substance that is found around the breast area, which can be seen through radiology. X-rays are modern ways of check-ups.

## Interviewee (30006)

[silence] It could be through radiology.

## Interviewee (300051)

The most important check-up is the radiology check-up. That is how the doctor knows whether there is a tumor or not.

## Interviewee (300066)

Then, he said that she had nothing, thanks Allah, and that he wanted her to go through a check-up via mammogram and also to do some blood analyses to reassure that she had nothing.

## Interviewee (800092)

I don’t know! It may be radiations. That is possible. But it is not any radiations. Definitely, they have a name but I don’t know what they are called.

## Interviewee (3000101)

After that, the doctor suggests that she goes through a check-up by radiations in order to examine what she has.

However, regarding the clinical check-up or the mammogram that is also called in French “Mammographie”, it is supposed to be done at least once a year; it is preferable to be done once per six months.

## Interviewee (6000131)

The next stage is to check on the breast by means of radiology to check if there is any malignant tumor or not. After that the folder of the patient is analyzed to make sure whether the patient is infected or not.

## Interviewee (6000135)

Last but not least, the third type of check-ups is the mammogram. That is the most perfect check-up known so far as it is very precise. It is the best technology out there up to now. Mammography allows the doctor to check on very microscopic substances that neither the woman nor the doctor can see. Such substances are usually located inside the breast and can’t be seen or felt by the doctor.

## Interviewee (6000136)

If there are any unusual substances showing around or inside the breast, the doctor transfers the patient to the third stage of check-ups that is radiology- it is more precise.

## The following 15 interviewees made no mention of mammography, radiology, x-ray examination, etc.

## Interviewee (200013)

Not specifically mentioned

## Interviewee (200085_n)

Not specifically mentioned

## Interviewee (600076)

Not specifically mentioned

## Interviewee (600077)

Not specifically mentioned

## Interviewee (6000150_n)

Not directly mentioned

## Interviewee (6000151)

Not directly mentioned

## Interviewee (100019_n)

Not directly mentioned

## Interviewee (600079)

Not specifically mentioned

## Interviewee (600083)

Not specifically mentioned

## Interviewee (2000154)

*Note: While filename is 200154, interviewee was 6000154)*

Not specifically mentioned

## Interviewee (6000146)

Not specifically mentioned

## Interviewee (6000148)

Not specifically mentioned

## Interviewee (6000132)

Not specifically mentioned

## Interviewee (6000133)

Not specifically mentioned

## Interviewee (6000134)

Not specifically mentioned

## NECESSITY OF BCSE

## The following 43 interviewees made specific mention of the necessity (or lack thereof) of BCSE.

Note: Highlighted in yellow are 2 interviewees who did not perceive that BCSE were necessary.

## Interviewee (100042)

yes, of course especially that the disease is widespread and this is something scary; so women should always take care of themselves to detect anything in early stages. She should not neglect herself and say this is something simple, or not very painful, she should do the examinations even if her doubts are not so big

I encourage women to do the examinations and especially the self-examination, it is very necessary and she should not neglect it. In the event she notices any changes, she should immediately go to the physician.

## Interviewee (100088_n)

yes, yes always, women should do the home test periodically for it is very important to detect the disease in its early stages and thus would be cured by God’s will. I advise all women to periodically check themselves in the medical center.

## Interviewee (200014_n)

Very important. The cancer, glory to God, spreads quickly if the patients do not take precautions against it since its early stages. It is a problem. One should do this early.

## Interviewee (200087)

the examinations are very important even if the women are not sure that they have cancer or even if they have nothing, they help us to be reassured and they are very useful. I advise any woman to do them because they are very important and they help all women to detect the breast cancer in early stages before it develops.

And it's a must for me and any other person to support woman in all domains, especially in this subject because it is a very dangerous matter and it is a disease which has no cure. It's a must to support her in this matter, because first of all it' s a matter of health.

## Interviewee (6000147)

: I support these examinations, not only for the breast but also for the vagina.

I encourage all women to do the X-ray imagery and all the examinations and not to neglect them especially when it comes to cancer because it is widespread; all women should be aware of these things and should always follow-up and check with physicians at hospitals.

## Interviewee (6000149)

they are of course necessary and required because it is a dangerous disease.

## Interviewee (6000151)

they are necessary

## Interviewee (100020)

I think that these examinations are very necessary and important since these examinations are widespread in our days so there is no problem that a woman goes and does the examinations. I encourage her. All people are subject to disease but it is better to detect diseases early and that’s why I do encourage examinations.

okay, I see and I encourage all women to do these examinations; frankly speaking, it is something very important and there is no shame about it, this is a disease from God.

## Interviewee (100050)

hey are necessary; I see that they are necessary

## Interviewee (100093)

these examinations are very important .... They are very important for the breast cancer detection.

## Interviewee (100097)

**Interviewer:** so do you support women to do the examinations or not?

Every person is free. But for me, for my mother and sisters, it is not necessary to do them. I am talking about my personal status. I have nothing to do with others. ... but if we don’t doubts about anything, this is not necessary.

## Interviewee (100098)

of course; more the examinations are, better the diagnosis is. A woman should do the three of them to be sure

## Interviewee (100081)

The examination are very important and the woman can do it.

## Interviewee (600075_n)

I feel that these examinations are beneficial if it is available in many places.

## Interviewee (200013)

I really hope that each and every Arab woman goes through a check-up. It does not heart and in the end it is a check-up that benefits her.

It is important that a woman goes through the three check-ups you mentioned earlier.

Well, I would like to encourage each and everybody to support the Arab woman to go through such check-ups. If the woman is not infected, she will stay away of it when she does the check-ups, anyways. As I told you, the check-ups allow one to know what their health status is. We need to maintain women healthy!

## Interviewee (200015)

So, I see that it is a must that the Arab woman should go through regular breast cancer check-ups.

Check-ups are vital not only for the own well-being of the woman but also for the good of her family and society in general.

## Interviewee (200085_n)

Well, it is very compulsory. Regular check-ups are very crucial because it helps discovering diseases at their early stages and also curing them before it is too late. I really would like to encourage people to go through Breast Cancer check-ups.

## Interviewee (200086)

Actually, I encourage all women, in case they feel a strange feeling around the breast area to go through medical examinations as check-ups at early stages prevents would prevent the woman of Breast Cancer.

## Interviewee (600018)

the least reaction is self-screening, and then visiting a doctor for reassurance.

## Interviewee (600074)

Hence, it is very important in that if there is something wrong, early screenings are beneficial to know the diseases since the beginning. Breast Cancer early detection definitely ends up with pleasant results.

In fact, these check-ups are very crucial. Precaution is better than cure.

Actually, these check-ups are very normal and it is vital that they should be done.

## Interviewee (600076)

The breast cancer check-up is essential. It is for their well-being.

## Interviewee (600077)

I think it is very important to both, the patient and the intact person.

## Interviewee (600078)

Yes, each and every woman has to go through breast check-ups.

## Interviewee (600079)

In a nutshell, the check-up must be general not specific. People don’t want to do specific check-ups. They would better go for a general check-up to know everything about their bodies.

## Interviewee (600080)

I see that it is a good thing. There is nothing wrong with it. It is, yet, good for the woman to make sure that she is healthy.

Check-up is important in general regardless of whether the woman is pregnant or not. However, check-up should be given more priority by the pregnant woman. If she is infected, she will definitely pass on the disease to her baby.

## Interviewee (600082)

I will definitely encourage her and follow up with her, myself, in all cases.

## Interviewee (600089)

Well, as I said before, it is a good thing. Early check-ups can result in the chance of a successful cure opportunity.

## Interviewee (2000153)

Early check-up is an essential step forward to discover abnormal cases or changes at the level of the breast tissue itself.

Women have to grasp, anyways, how important breast cancer check-ups are to avoid picking up the disease.

## Interviewee (2000154)

*Note: While filename is 200154, interviewee was actually 6000154)*

Of course, I do encourage breast cancer check-ups. A check-up can save the life of a person.

## Interviewee (6000146)

In fact, they are good. One is obliged to go through check-ups for precaution reasons.

## Interviewee (10005_n)

Absolutely! When the woman feels that there is something wrong, she has to keep following up on the consultation until she makes sure that nothing is wrong at all and that her life is 100 per cent safe.

## Interviewee (30004)

So, as we said, the clinical check-up is compulsory.

This is something not to argue about. If she has just 1% of doubt, the woman has to consult a doctor. She even should not wait until she notices symptoms. The woman has to consult a doctor without necessarily feeling anything. Check-ups are important.

The solution to breast cancer is medical check-ups. That is it.

## Interviewee (30006)

The check-ups are very vital. It is very important that the woman goes through the check-ups. The disease cannot be figured out if she is not checked up. They are also important because they prevent the development of the disease and its spread over in the human body. In the end, it is a tumor. When checked up at early stages, it gives big chances that the patient would be totally cured of it. It will be so easy to eradicate it.

## Interviewee (300051)

That is a must! Such check-ups should be undergone without even feeling anything. Such check-ups can be done and the results are not necessarily negative.

## Interviewee (300066)

Of course they are so important. What if that substance that was in the breast of my wife was a cancer and we did not consult a doctor? That could have caused so many problems.

## Interviewee (800092)

I would love to give advice to women but I don’t like to put pressure on them. Just the words “breast cancer” are big words. Personally, I don’t want to do much research about it because if I do that, I will be always living in delusion. I will be always thinking about it and always afraid that it infects my family.

I would encourage my wife to go through a check-up in order to catch it earlier if there is anything wrong, May Allah forbid!

If my wife and I, both, witness the same symptoms, then I will encourage her to go through a check-up. Yet, if there isn’t anything, she should not bother consulting a doctor.

If the symptoms are clear and there were more than one, I would encourage her to go through these check-ups.

## Interviewee (3000101)

In my opinion, these check-ups are very compulsory.

## Interviewee (6000131)

Definitely yes! It is one of the most important and dangerous check-ups that should be undergone especially for women.

## Interviewee (6000132)

That is very important. It is such a crucial and vital thing to do. Society is so in need of such check-ups in order to stop the widespread of this disease.

## Interviewee (6000133)

Women should be encouraged by each and every one in the family to do these check-ups.

## Interviewee (6000134)

Women need to be encouraged. They need to be aware of the fact that Breast Cancer check-up is like any other regular check-up.

## Interviewee (6000135)

In my opinion, the check-up is very important. Through check-ups, women can tell whether they are infected or not; and if infected, can tell whether the degree of danger is high or not.

## Interviewee (6000136)

In my opinion, this check-up is very compulsory for women. What I know it that check-up on breast cancer at an early stage helps better in the treatment. I also know that tumors that appear at the very first stage of the cancer are not dangerous. However, if left without treatment, they alter to become malignant. Therefore, early stage check-ups are very vital.

## The following 7 interviewees made no specific reference to the necessity of BCSE although some did mention regular examinations.

## Interviewee (6000150_n)

Not directly mentioned

## Interviewee (100019_n)

Not directly mentioned

## Interviewee (100099)

Not directly mentioned

## Interviewee (1000100_n)

Not directly mentioned

## Interviewee (200084)

Not specifically mentioned

## Interviewee (600083)

Not specifically mentioned

## Interviewee (6000148)

Not specifically mentioned

## REGULARITY OF PERFORMING BCSE

## The following 45 interviewees made specific reference to regular BCSE.

## Interviewee (100042)

She should be always aware and should do the examinations at least once per month.

if she is young and didn’t feel anything, she could visit the physician once per year

If things are normal, it is sufficient to do the examinations once per year for more reassurance. Sometimes the disease is present but does not appear since its early stages so the annual clinical examination is a mean to detect the disease in early stages and this is better. But if the woman notices something, she should immediately go to the physician even if it is not cancer necessarily or even if it is any other disease. The rumor may not be cancer but the importance is to be reassured. The annual examination is good.

Old women should do continuous examinations; after the menopause, they should do constant examinations and should not be based only on the self-examination. They should always visit the physician at least once per year to do the necessary examinations.

## Interviewee (100088_n)

I even encourage women to check themselves periodically without even feeling any of the disease’s symptoms to detect the disease in its early stages

## Interviewee (200014_n)

In fact, the woman should every six months go for examination.

Yes. If she feels any tumor or notices any abnormal thing, she goes for examination every 6 months or something like that.

No, it is not necessary to have a tumor. She goes, I mean it is a routine examination.

**Interviewer:** So it is better to go to the hospital for examination on a regular basis than doing a self-examination at home even if she is experienced with this kind of examination.

**Interviewee:** Yes, yes

## Interviewee (200087)

**Interviewer:** do you think Arab women need to do the breast cancer examinations: whether the self-examination, the mammogram or others on a regular basis or not?

sure, but not only for Arab women as you said but for all women in general. If a woman is infected with breast cancer without being aware of that and is breastfeeding her baby at the same time, this could be dangerous for her baby and this disease could pass to her baby; this is something very dangerous. She is putting in danger her life of her baby; that’s why it is better for her to do the examinations to be reassured about her health and her babies’ health and to be sure that they won’t be infected with the same disease.

## Interviewee (6000147)

Everywoman should take care of herself and take the necessary prevention measures if not every six months, at least every one year.

yes, yes, this is something very necessary and important. My wife does examinations every six months, I always tell her she should do them every six months

## Interviewee (6000149)

Every woman could do the examination at home and then go at least once every six months to do the examinations at the hospital.

## Interviewee (6000150_n)

**Interviewer:** okay. Do you encourage women to do a routine examination every six months or one year?

of course I do; since this disease is widespread, I of course encourage them to do the examinations as long as it is available and easy

## Interviewee (6000151)

they are necessary and should be performed regularly; any woman or man should do regular examinations, every six months or once per year. So these are important to detect the disease since early stages. I hear that if this disease is detected in early stages, it could be easily treated. This is what I know

## Interviewee (100019_n)

She should do periodically control and do blood test every six months or every year which is recommended so it can be treated in its early stage.

## Interviewee (100020)

**Interviewer:** so women need to do these examinations on a regular basis

yes, of course, this is a dangerous disease

**Interviewer:** how should women do it?

I don’t know the steps exactly, as you said there are the self-examination performed by the woman herself, the clinical examination performed by specialists and the imagery; so I encourage women to do them all

**Interviewer:** okay, you encourage women but my question was: do Arab women need to do the examination on a regular basis?

yes hey need to do them

on a regular basis because this disease could appear at any time so you may go and do the examinations today and you have nothing; however, this disease could appear in one week.

## Interviewee (100093)

women should do the mammogram yearly

## Interviewee (100097)

I know that the woman should do periodical examinations every six months. She should get examined. There is nothing bad about it since it is a normal examination. It is ordinary and the woman should examine herself to be reassured about her health.

no. no. it is possible to teach the woman how to perform the self-examination and she can do it whenever she wants. But it is so difficult that a woman goes and gets examined by a physician, unless the physician was a female, she can do the clinical examination every two or three years.

**Interviewer:** I will repeat my question to you: do you think Arab women need to do the breast examinations on a regular basis?

no, they don’t need to do them unless a woman feels like to examine herself. But to go and get examined by a physician, no.

**Interviewer:** she may need to do the clinical examination

but not on a regular basis like every 6 months; she could do the self-examination every month, every two months or three months, I am not obliged to do it on the 2^nd^ of each month, she could do it on the 6^th^ or the 7^th^. There is no defined time, she can do it every one month, two, three, four, five or six months. And it is always about the self-examination while taking a shower, a bath, okay. However, if she knows that she has cancer, she goes to the physician but she doesn’t let him examine her, she does the X-ray imagery. If the X-ray proves that she has cancer, she could go to a physician, a female physician f course, not a male physician. A male physician is the last resort, last phase.

**Interviewer:** we will go back a little bit to the beginning to your opinion about the examinations. In your opinion, women do not need to do the examinations on a regular basis

true

**Interviewer:** so do you support women to do the examinations or not?

And this is unless with have doubts that she has a certain disease, she could do a periodical examination every four or six months; but if we don’t doubts about anything, this is not necessary.

## Interviewee (100098)

yes, of course she needs to do them on a regular basis for the prevention of this disease or to detect it early since the treatment chances are higher when detected early. I guess it would be easier for the physician to treat the disease and to limit damages

## Interviewee (100099)

it is better for the woman to do the examinations on a regular basis – if there is a medical case in the family.

## Interviewee (1000100_n)

I understand what you mean. We the Arabs don’t go to the physician unless when we have a problem. But doing a periodic examination, this is not applicable in our culture....

I will tell you something. We are talking about women now, when a woman has a good financial condition, she should do a periodical examination; when money is available and the means are available, she should do the examinations to maintain her good health, to remain beautiful, agile, active and in a perfect state. Maybe women here are doing periodical examinations, here women do the periodical examinations. The answer is not clear?

## Interviewee (200084)

**Interviewer:**  Ok! Do you think that the Arab woman should go through regular check-ups, be they self-check-ups or consulting a doctor?

Generally speaking, it is preferable. If check-ups are beneficial for her and not expensive, why should not she go through them?

## Interviewee (200013)

Well yes! The check-ups must be regular at least once a year. That is very important.

## Interviewee (200015)

One needs to catch up the check-up since the beginning by going through regular and periodic check-ups.

## Interviewee (200085_n)

I encourage periodic check-ups a big deal.

## Interviewee (200086)

Definitely, I support the idea that women have to go through regular inspections. Early examinations of Breast Cancer make the treatments easier and save them money as well.

## Interviewee (600018)

Regular check-up is very crucial and there is nothing to lose in doing it.

It is important that check-ups are periodic and regular. It is so crucial that such check-up are conducted on regular basis, especially radiology.

## Interviewee (600074)

It should be done on regular basis. The person has to doctor themselves. The woman has to do a self-screening. In case she finds out a strange element in the body, a light tumor or feels a pain, she should not keep it hidden; however, she automatically has to go through rigorous check-ups that indicate whether there is a disease or not.

## Interviewee (600077)

Not going through check-ups regularly is the main cause of Breast Cancer.

The check-up should be done every one year or six months. That depends on the age of the person.

Well, the old mentality is that people don’t consult a doctor unless they are sick. The new mentality is that people consult doctors once per 6 months or year to avoid picking up diseases.

## Interviewee (600078)

Therefore, everyone has to go through breast cancer check-ups in a regular manner.

## Interviewee (600079)

When one goes over the age of forty, regular check-up has to become a must.

## Interviewee (600080)

One has to go through check-ups regularly, especially women.

Out of fear that something bad happens to them, I would encourage them to go through regular check-ups every now and then.

## Interviewee (600082)

Also, there be should be a regular check-up with a doctor each 6 months.

I think that women have to do the check-ups each 6 months to catch up the diseases at its first stages and follow the treatment

## Interviewee (600083)

I do encourage women who go through check-ups. I really do encourage them! *(talking about checkups in general, not necessarily BCSE)*

## Interviewee (600089)

Well, yes, without a doubt! This is something that should be spontaneous in a women’s life. To ensure protection, women have to go through regular check-ups.

For preventions reasons, I would definitely encourage my wife to go through regular check-ups because when things are discovered early, the cure is not usually complicated and even the side effects are almost absent.

## Interviewee (2000153)

I wish that every Arab woman has awareness that periodic check-ups are very important in order to avoid breast cancer infection.

## Interviewee (2000154)

*Note: While filename is 200154, interviewee was actually 6000154)*

If the check-up is done regularly, people will avoid being infected by many diseases.

## Interviewee (6000146)

Regarding the regular check-up, this is something important. One has to go through check-ups.

## Interviewee (6000148)

It is preferable that one goes through a check-up each two or three months. The check-up shall not be on breast cancer only.

As far as I am concerned, I encourage the idea that women go through regular check-ups at least every other month. *(Speaking of checkups in general, not necessarily BCSE)*

## Interviewee (30004)

The danger that breast cancer represents calls for regular check-ups following the three steps we have discussed earlier. The thing is that the girl or the woman checks on herself first, then consults a doctor and if there is a need, she goes through a mammogram diagnosis.

In addition to that, women have to go through these check-ups twice or at least once a year.

## Interviewee (30006)

Women have to go through regular and continuous bases each six months. It is a must that the woman goes through such check-ups. Here comes the role of the husband. It is up to him to encourage his wife to undergo regular check-ups

## Interviewee (300051)

Regular check-ups are a must.

Yes, these check-ups could be done on monthly bases.

## Interviewee (300066)

It is not necessary that she goes regularly. One or two times per year are enough unless she feels that there is something wrong going on with her body. She could even consult a doctor once per month.

The experience that I have been through taught me that one should undergo regular check-ups.

## Interviewee (800092)

Doing these check-ups regularly will cause delusions to the woman. I prefer that it should not be so regular.

## Interviewee (3000101)

That should be done at least once a year.

**Interviewer:**

Do you mean many times per year?

No, I mean once a year. If the woman wants to protect herself, she has to go through a check-up at least once a year. Regarding the self-check-up, this could be done every now and then. It shall not be tied to a specific period of time. However, regarding the clinical check-up or the mammogram that is also called in French “Mammographie”, it is supposed to be done at least once a year; it is preferable to be done once per six months.

## Interviewee (6000131)

Such check-ups should be done regularly and if not regularly, one has to check up in case something abnormal is felt. This applies to both single girls and married women and that is after the parental accord of course. ... So we are obliged to do regular check-ups and even our religion asks us to take care of ourselves and do check-ups regularly.

So as I said women, either Arab or non-Arab, have to do regular check-ups. Unlike Arab and Muslim women, western women have to pay more attention to undergoing check-ups as they are ethically and morally unbounded. But, in general, people have to go through regular check-ups.

## Interviewee (6000132)

Well, I believe that women should consult doctors every now and then. It is up to the doctor in the medical center to determine whether the period of the check-up is three or six months or one year, etc.

## Interviewee (6000133)

As far as Breast Cancer is concerned, women are usually more susceptible to it. That is why they should go through regular check-ups every three or six months, up to one year.

Of course, yes! In order for her to protect her life and her family, she has to go through regular check-ups.

## Interviewee (6000134)

The thing is that there must be check-ups on regular bases.

It is compulsory that one goes through a full check-up every 6 months or at least once year.

## Interviewee (6000135)

Of course, that is for granted. This question is repeating and I will give you the same answer: check-ups are very important to stop the spread out of the disease in Qatar or any other Arab state.

## Interviewee (6000136)

As I said before, regular check-up is very important. It helps a lot in the treatment process. In order to figure out whether there is a cancer or not, the Arab woman has to do a regular check-up on her breasts in order to avoid the development of the tumor, which makes it so hard to control the virus not to spread all over the body.

## The following 5 interviewees made no specific comment regarding the necessity of regular BCSE.

## Interviewee (600076)

## Interviewee (100050)

## Interviewee (100081)

## Interviewee (600075_n)

## Interviewee (10005_n)

# AGE FACTOR

## The following 19 interviewees specifically mentioned age as a factor in BCSE

## Interviewee (100042)

women after the menopause should do regular examinations because they are subject to this disease more than the others so focus should be made on old women more than young women. So old women should never neglect the examinations even if nothing is detected from the self-examination, they should go to the physician

An old woman skin is thicker and she may detect nothing by palpation when performing the self-examination although she should not be based only on the self-examination but should see as well the physician on a regular basis, at least once per year. As for younger woman, 30-year old women may – if they are doing the self-examination – postpone the physician visit on condition that nothing is detected unlike old women after the menopause.

## Interviewee (200014_n)

I mean it is difficult, it is difficult in particuar for women who are 40 year-old and more, this may be applicable for those at the age of 30, but women at the age of 40 do not care about this matter.

## Interviewee (6000150_n)

of course, a 20-year old woman is different that a 50-year old woman

a 20-year old woman may read newspapers, be aware of new things, check things on internet but old women may be a little bit far from these things

## Interviewee (100098)

As for the necessary examinations, women after the age of 40 shall do periodical examinations because of the age factor and the genetic factor that plays a great role in breast cancer.

## Interviewee (1000100_n)

women after the age of 40 should do a periodic examination because after the menopause, she will experience many changes and thus she should go to the specialists because this disease early detection allows us preventing a lot of symptoms that could infect women later on

## Interviewee (600075_n)

such examination should started from the age of puberty 12 or 13 years old.

## Interviewee (600018)

So, once the woman reaches the age of 35 years, she needs to start doing checks up in order to stop it in case it is at its early stages; and therefore, get rid of it.

## Interviewee (600077)

If the woman is fifty, she has to go through a check-up each three or six months. However, younger people can do it every six month or one year. That is something crucial.

## Interviewee (600079)

It is preferable that women above the age of forty go through regular check-ups. An early check-up is good for many reasons.

So, if we want to encourage women to go through check-ups, they have to be provided with a general clinic where women after the age of forty go through the check-up.

## Interviewee (600082)

the symptoms of Breast Cancer appear after the age of forty. It is usually between forty and fifty.

## Interviewee (600089)

It is known that Breast Cancer targets women at their menopausal age. It is compulsory that the check-up is undergone regularly so that the possibility of cure is higher in case there is any infection.

## Interviewee (2000153)

At some ages, it is not really necessary to go through such check-ups.

At a certain age, the woman has to go through this check-up.

## Interviewee (2000154)

*Note: While filename is 200154, interviewee was actually 6000154)*

Well, I so encourage women who surpassed a certain age to go through such check-ups.

## Interviewee (6000146)

To the best of my knowledge, women after the age of forty should start going through such check-up on regular bases.

## Interviewee (800092)

I hear that it happens to old people not to youngsters

I guess that it targets people over the age of fifty*. (not specifically talking about older women doing BCSE)*

## Interviewee (300066)

No matter how old she is, the woman has to go through these check-ups from time to time.

## Interviewee (6000132)

Well, what I know is that Breast Cancer happens to women who are forty years old and above. *(not specifically about performing BCSE)*

## Interviewee (6000135)

This disease appears with women at the age of 50 and this does not mean that it can’t appear on women at an earlier age. *(not specifically about performing BCSE)*

## Interviewee (6000136)

I think that it happens at the age of fifty and above. It may also happen to younger women but as far as I know is that it targets elderly women. . *(not specifically about performing BCSE)*

The (female) doctor also advised my wife that she should do the check-up at the age of 40 and above even without noticing Cancer symptoms.

## The following 31 interviewees did not directly refer to an age factor

## Interviewee (100088_n)

## Interviewee (200087)

## Interviewee (6000147)

## Interviewee (6000149)

## Interviewee (6000151)

## Interviewee (100019_n)

## Interviewee (100020)

## Interviewee (100050)

## Interviewee (100093)

## Interviewee (100097)

## Interviewee (100099)

## Interviewee (100081)

## Interviewee (200084)

## Interviewee (200013)

## Interviewee (200015)

## Interviewee (200085_n)

## Interviewee (200086)

## Interviewee (600074)

## Interviewee (600076)

## Interviewee (600078)

## Interviewee (600080)

## Interviewee (600083)

## Interviewee (6000148)

## Interviewee (10005_n)

## Interviewee (30004)

## Interviewee (30006)

## Interviewee (300051)

## Interviewee (3000101)

## Interviewee (6000131)

## Interviewee (6000133)

## Interviewee (6000134)
